# Single nucleus transcriptomics reveals ITGA8-driven fibroblast remodeling in rheumatic valvular atrial fibrillation

**DOI:** 10.1016/j.isci.2026.117310

**Published:** 2026-09-01

**Authors:** Jinjin Wu, Yuliang Wang, Weizhuo Liu, Zhengjie Wang, Jingjing Liu, Zikun Wang, Lei Yang, Lin Sun, Yilin Pan, Yangyang Sun, Li Zhang, Yongjun Qian, Yangyang Zhang

**Affiliations:** 1 Department of Cardiology, Shanghai Children’s Medical Center, Shanghai Jiao Tong University School of Medicine, Shanghai, China; 2 The First Affiliated Hospital of Zhejiang University School of Medicine, Hangzhou, China; 3 Department of Critical Care Medicine, Shanghai Chest Hospital, Shanghai Jiao Tong University School of Medicine, Shanghai, China; 4 Department of Cardiovascular Surgery, West China Hospital, Sichuan University, Chengdu, China; 5 Department of Cardiology, Wuxi People’s Hospital Affiliated to Nanjing Medical University, Wuxi, China; 6 Department of Cardiovascular Surgery, Shanghai Chest Hospital, Shanghai Jiao Tong University School of Medicine, Shanghai, China; 7 The First Clinical Medical College of Nanjing Medical University, Nanjing, China; 8 Shanghai Institute of Precision Medicine, Shanghai Jiao Tong University School of Medicine, Shanghai, China; 9 Department of Vascular Surgery, The General Hospital of Ningxia Medical University, Yinchuan, Ningxia, China

**Keywords:** rheumatic valvular AF-associated remodeling, single nucleus RNA-sequencing, cardiac fibrosis, macrophages

## Abstract

Atrial fibrillation (AF) associated with rheumatic valvular disease involves complex cellular remodeling of the atrial myocardium. Here, single-nucleus RNA sequencing of left atrial tissues from healthy donors and patients with persistent rheumatic valvular AF identified disease-associated transcriptional changes across major cardiac cell populations. Fibroblasts showed marked heterogeneity, with expansion of an ITGA8-enriched FB-C2-ELN state associated with extracellular matrix remodeling. Functional experiments demonstrated that ITGA8 promotes cardiac fibroblast proliferation, migration, and matrix production. In an ACh/CaCl2-induced rat model, fibroblast-targeted ITGA8 suppression reduced atrial fibrosis and AF susceptibility. These findings define cellular states associated with atrial remodeling and highlight fibroblast ITGA8 signaling as a regulator of fibrosis-related AF progression.

## Highlights

• Single-nucleus RNA-seq maps atrial cell states in rheumatic valvular AF
• An ITGA8-enriched FB-C2-ELN fibroblast state expands in rheumatic valvular AF
• ITGA8 promotes fibroblast proliferation, migration, and extracellular matrix production
• Fibroblast-targeted ITGA8 knockdown reduces atrial fibrosis and AF susceptibility

## Graphical abstract

**Figure:**
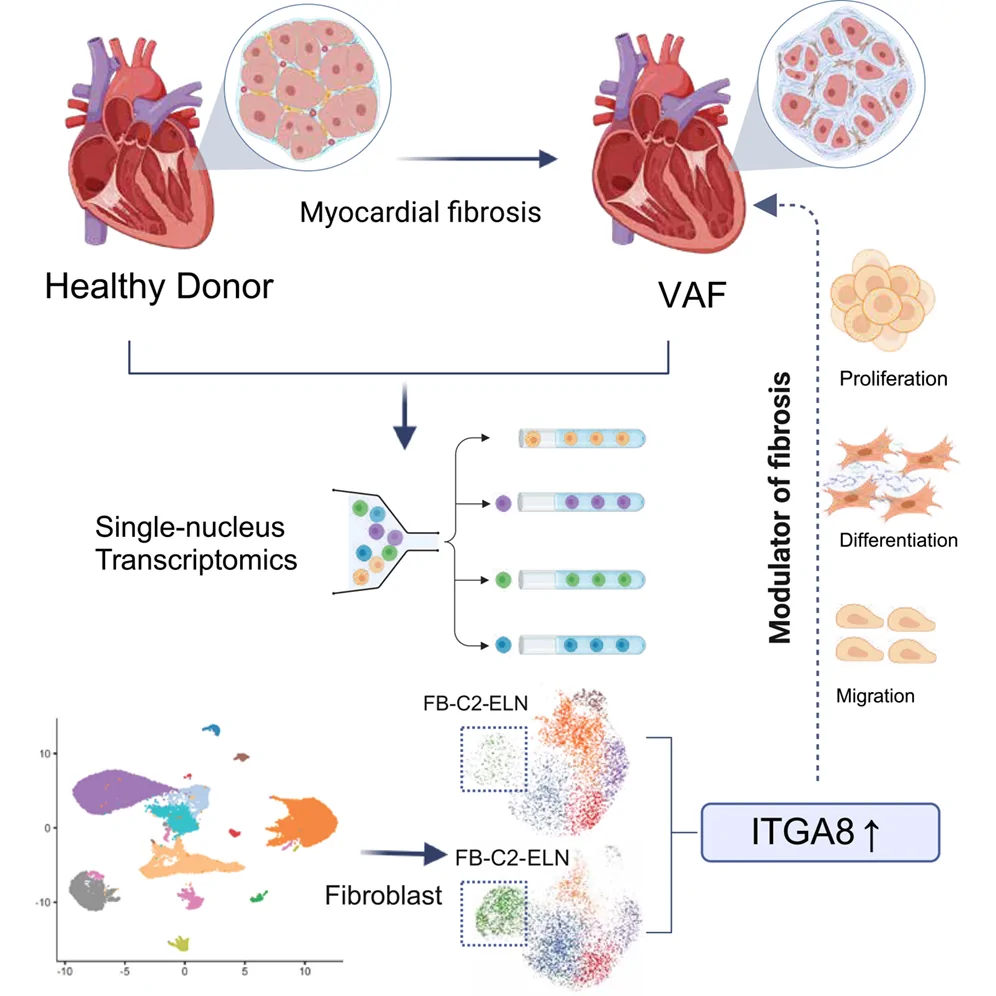

## Teaser

Health sciences

## Introduction

Single-cell RNA sequencing (scRNA-seq) and single-nucleus RNA sequencing (snRNA-seq) technologies have emerged as important research tools for the identification of distinct cell types in both healthy and diseased human tissues, and thus help us in understanding the pathological mechanisms of diseases more deeply.^1^^,^^2^^,^^3^ The advent of single-cell sequencing technologies has revolutionized our ability to dissect cell populations in more details and uncover specific disease-related cell states across all human organs.^4^^,^^5^ The rapid development of these powerful technologies significantly enhances our capacity to study complex biological systems at the single-cell level, which provided valuable insights into the heterogeneity of cell populations and their functions in diverse tissues and disease states.

Atrial fibrillation (AF) represents one of the most commonly occurring arrhythmic disorders. AF is associated with thrombosis, stroke, and heart failure, leading to a significant rise in both mortality and disability.^6^ The incidence of AF is estimated to be approximately 1%–2% in adults, with a much higher incidence of up to 12% in elderly populations.^6^ Due to the anticipated rising number of AF patients with an increasingly aging population, it is vital and urgent to clarify the pathogenic mechanisms underlying AF disease, providing a novel mechanism and therapeutic target for AF.

In recent years, snRNA-seq technology has been successfully implemented in healthy and diseased hearts, including heart failure, dilated cardiomyopathy (DCM), and other cardiovascular diseases (CADs), and help us in deeply disclosing the composition of cardiac cell populations and disease-specific subpopulations.^7^^,^^8^^,^^9^^,^^10^^,^^11^ These snRNA-seq studies have revealed significant cellular diversity, not only in cardiomyocytes (CMs) but also in non-myocytes, including endothelial cells (ECs), fibroblasts, myeloid cells, and neural cells.^8^ It has been reported that the transcriptional profile of atrial cardiac cells is significantly different from ventricular cardiac cells.^12^ However, there is limited knowledge of the cellular diversity and transcriptional signatures of human cardiac cell populations in the human atrial and the application of single-nucleus sequencing technology to human AF hearts is lacking.

In this study, we applied snRNA-seq technology to resolve single cell transcriptome in human cardiac samples obtained from healthy subjects and patients with valvular AF (VAF). Our analysis revealed 12 major cardiac cell types in human atrial and disclosed the diversity within each population. More importantly, we found that certain cell types exhibited VAF-related gene expression patterns, suggesting that the VAF-specific cell type may play a role in the development and progression of this disease. Our results offer a valuable resource for researchers and doctors to disclose the cellular biology of VAF in humans. By providing detailed information about the gene expression profiles of different cardiac cell types, we have shed light on the molecular and cellular mechanisms underlying VAF. Furthermore, our results have identified potential VAF-associated targets which may ultimately lead to the development of novel treatments for VAF.

Because atrial fibrosis and electrical remodeling provide the substrate for AF maintenance and for the “AF begets AF” phenomenon, functional perturbation of disease-relevant regulators is essential to distinguish candidate markers from causal mediators.^13^^,^^14^ ACh-CaCl2-induced rat models have been widely used to reproduce AF susceptibility driven by autonomic activation, calcium overload, and atrial remodeling.^15^^,^^16^^,^^17^^,^^18^ Thus, to complement our human snRNA-seq atlas and fibroblast assays, we further introduced an Adeno‑associated virus 9 (AAV9)-based ITGA8 intervention in this model to evaluate whether modulation of the ITGA8-related remodeling program could affect cardiac function and AF vulnerability *in vivo*.

## Results

### Isolation and analysis of the cardiac cellulome by snRNA-seq

**Figure 1:**
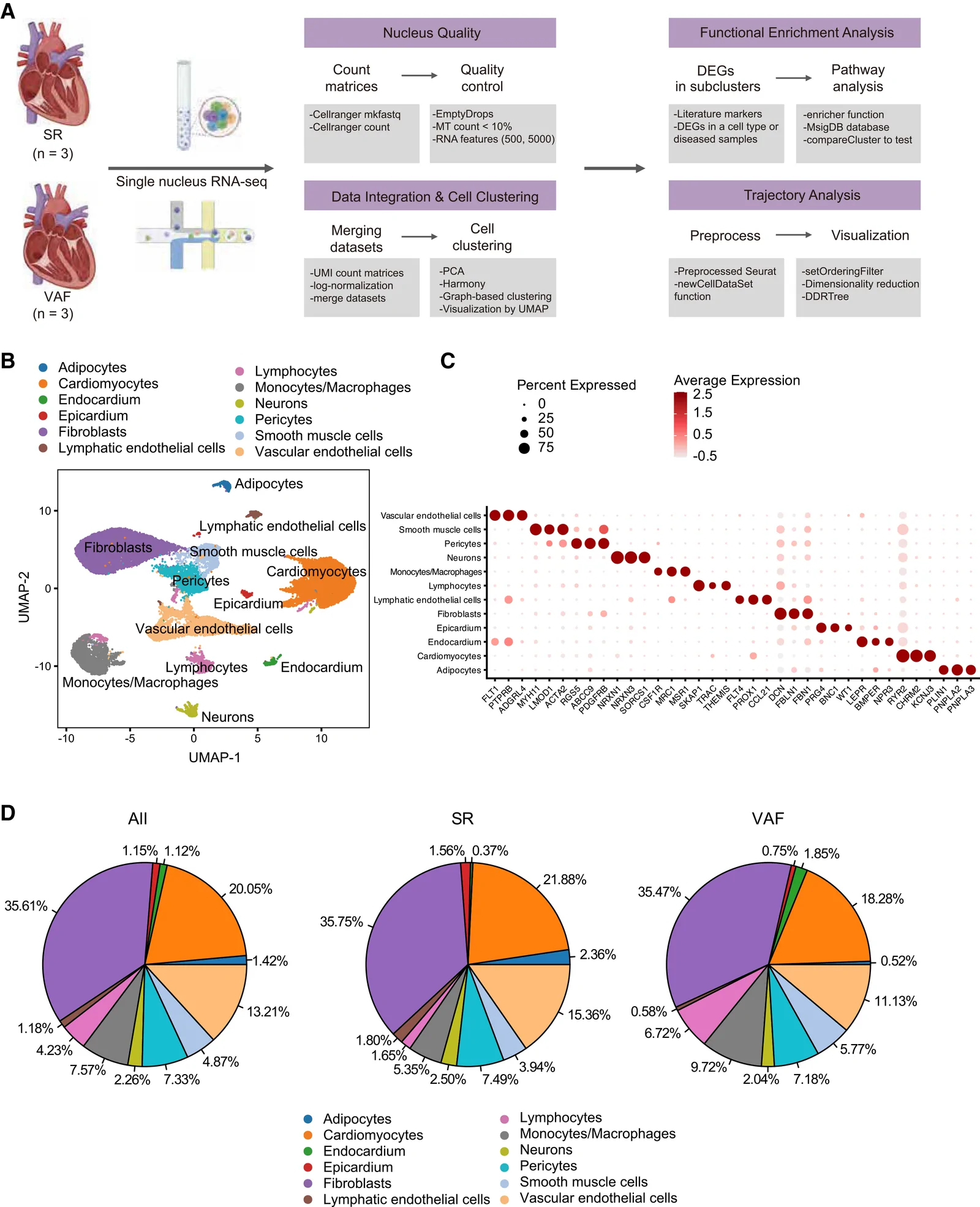
Cellular composition of the healthy and VAF human heart (A) Schematic representation of the design of the snRNA-seq experiments. Transmural sections were obtained from the left auricular appendage during donor heart procurement for sex and age comparisons (snRNA-seq, *n* = 3 SR, *n* = 3 VAF). (B) Unsupervised uniform manifold approximation and projection (UMAP)-based dimensional reduction of 37,425 nuclei after QC and data filtering. (C) The marker genes of the cell types identified by snRNA-seq. The color represents the average expression and the point size represents the percent of cells expressed the marker genes. We used FLT1, PTPRB, and ADGRL4 as vascular endothelial cell markers; MYH11, LMOD1, and ACTA2 as smooth muscle cell markers; RGS5, ABCC9, and PDGFRB as pericyte markers; NRXN1, SORCS1, and NRXN3 as neuron markers; CSF1R, MRC1, and MSR1 as monocyte/macrophage markers; SKAP1, TRAC, and THEMIS as lymphocyte markers; FLT4, PROX1, and CCL21 as lymphatic endothelial cell markers; DCN, FBLN1, and FBN1 as fibroblast markers; PRG4, BNC1, and WT1 as epicardium markers; LEPR, BNMPER, and NPR3 as endocardium markers; RYR2, CHRM2, and KCNJ3 as cardiomyocyte markers; PLIN1, PNPLA2, and PNPLA3 as adipocyte markers. (D) The pie charts showing the proportion of cells within all 6 heart samples, 3 healthy and 3 VAF heart samples. Our study integrated data from both healthy and VAF patients.

Left atrial appendage (LAA) cardiac tissue specimens from 3 non-diseased donors (sinus rhythm [SR] group) and 3 individuals with VAF (VAF group) were obtained to define the cellular and transcriptional landscape of the SR and VAF human heart. Baseline characteristics were shown in Table S1. Transmural myocardial samples from LAA were then processed for snRNA-seq (*n* = 6) using the 10*×* Genomics 3ʹ Single Cell platform. Single-nucleus libraries were sequenced, aligned to the human reference genome, and filtered for quality control (QC), after which unsupervised clustering, integration, and differential expression analysis were performed using Harmony and Seurat (Figure 1A). Our study integrated data from both healthy and VAF patients. After QC filtration, we obtained a total of 37,425 nuclei (Figures S1A–S1C), and VAF samples showed less unique molecular index (UMI) and feature count than the SR samples (Figures S1B and S1C, Wilcoxon test, *p* < 0.001). The final integrated dataset consisted of 37,425 nuclei representative of 12 major cell types (Figure 1B), including fibroblasts, CMs, monocytes/macrophages, pericytes, vascular ECs, smooth muscle cells, lymphocytes, neurons, adipocytes, lymphatic ECs, endocardium, and epicardium.

Cell identities were determined by the cell-specific expression of marker genes (Figure 1C). As shown in Figure 1D, amongst all major cell types in the whole population, fibroblasts accounted for the highest proportion (35.6%), followed by CMs (20.0%) and ECs (13.2%). The percentages for monocytes/macrophages were 7.6% (Figure 1D). Since fibroblasts, CMs, ECs, and monocytes/macrophages compose the highest four populations in human hearts, we chose to focus our analysis on how VAF influences these major cardiac cell populations.

### Subclustering of fibroblasts and subsequent analysis of cardiac fibrosis-related gene expression

**Figure 2:**
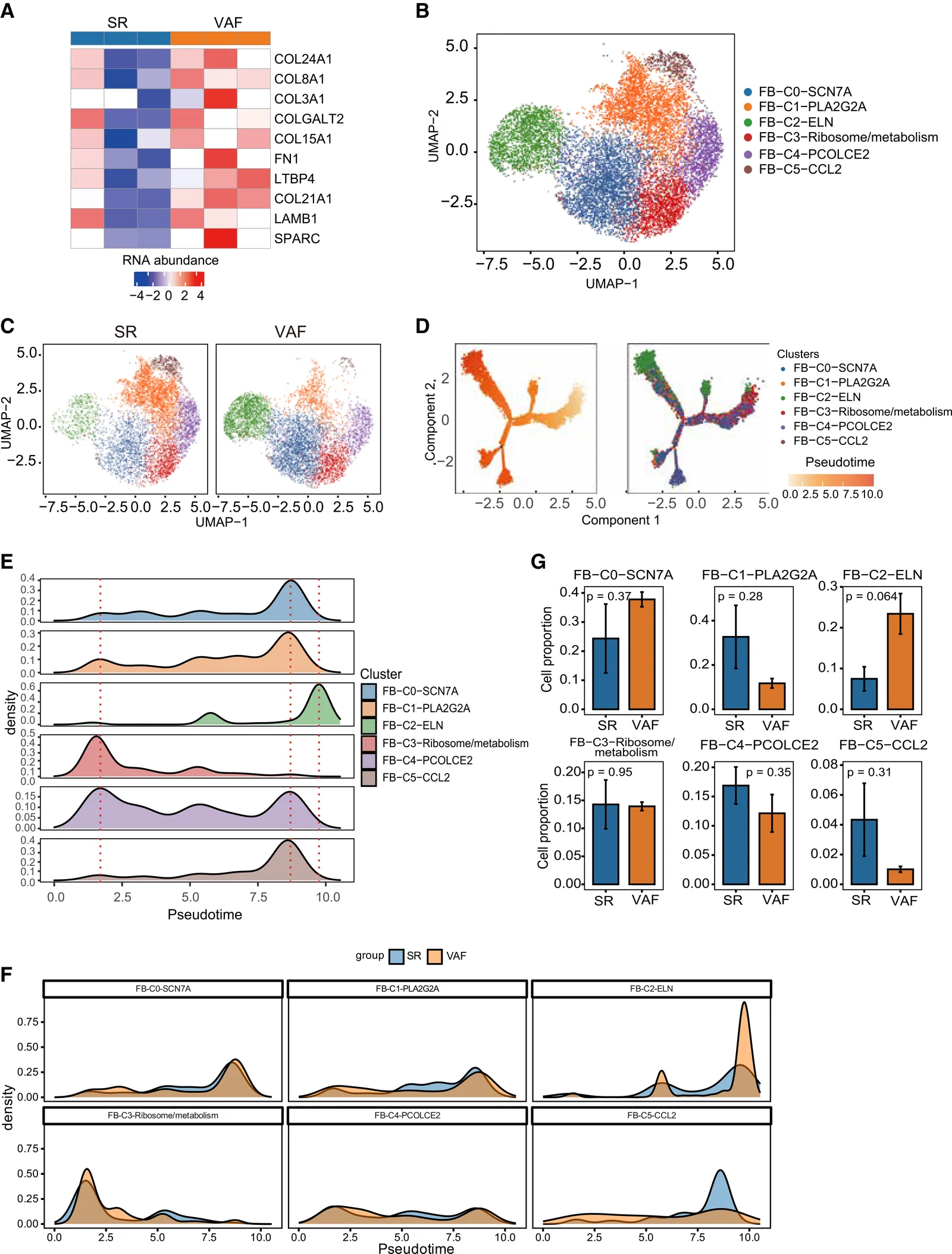
VAF-related cardiac fibroblast subsets by subclustering analysis (A) The expression patterns of ECM-related genes within the cardiac fibroblasts of VAF and SR. The gene expression levels of each sample are obtained by calculating the average expression values of all cells within each sample. The average expression levels of the six samples were normalized to *Z* scores. (B) The subclustering of fibroblasts identified six functionally distinct subsets of cardiac fibroblasts. The cardiac fibroblasts were visualized by UMAP dimensional reduction method, and the colors represent the six fibroblast subsets, respectively. (C) Distribution of the six fibroblast subsets within SR (left) and VAF (right). (D) Monocle2 trajectory analysis of fibroblasts colored by pseudotime (left) and fibroblast subsets (right). (E and F) The density curves of different subsets of fibroblasts over the pseudotime. (G) Bar plots showing the proportion of six fibroblast populations in SR (blue) and VAF (orange) fibroblasts. Data were shown as mean ± S.E.M. The *t* test was used for comparisons between two groups.

Gene differential expression analysis revealed that many extracellular matrix (ECM)-related genes (COL24A1, COL8A1, COL3A1, COLGALT2, COL15A1, FN1, LTBP4, COL21A1, LAMB1, and SPARC) were significantly upregulated (false discovery rate [FDR]-adjusted *p* < 0.05 [and |log2FC| > 0.5] [fold change]) in VAF samples compared to SR (Figure 2A). Unsupervised clustering of the integrated dataset revealed six distinct populations of fibroblasts (Figures 2B and 2C). We categorized these subclusters based on their transcriptomic fingerprints and alignment with established cardiac fibroblast atlases. Specifically, two major populations primarily observed in SR were identified: fibroblast (FB)-C0-SCN7A, characterized by the top-ranking marker SCN7A, and FB-C1-PLA2G2A. (Figures 2B and 2C). We observed additional minor fibroblast subpopulations including FB-C2-ELN, FB-C3-ribosome/metabolism, FB-C4-PCOLCE2, and FB-C5-CCL2 (Figures 2B and 2C). Notably, the expression profiles of FB-C1, C2, C4, and C5 showed high fidelity to the fibroblast states (Fb4, Fb5, Fb2, and Fb7, respectively) previously described by Koenig et al.,^7^ whereas FB-C3 was uniquely defined by its enriched ribosomal and metabolic programs rather than a single canonical marker.

**Figure 3:**
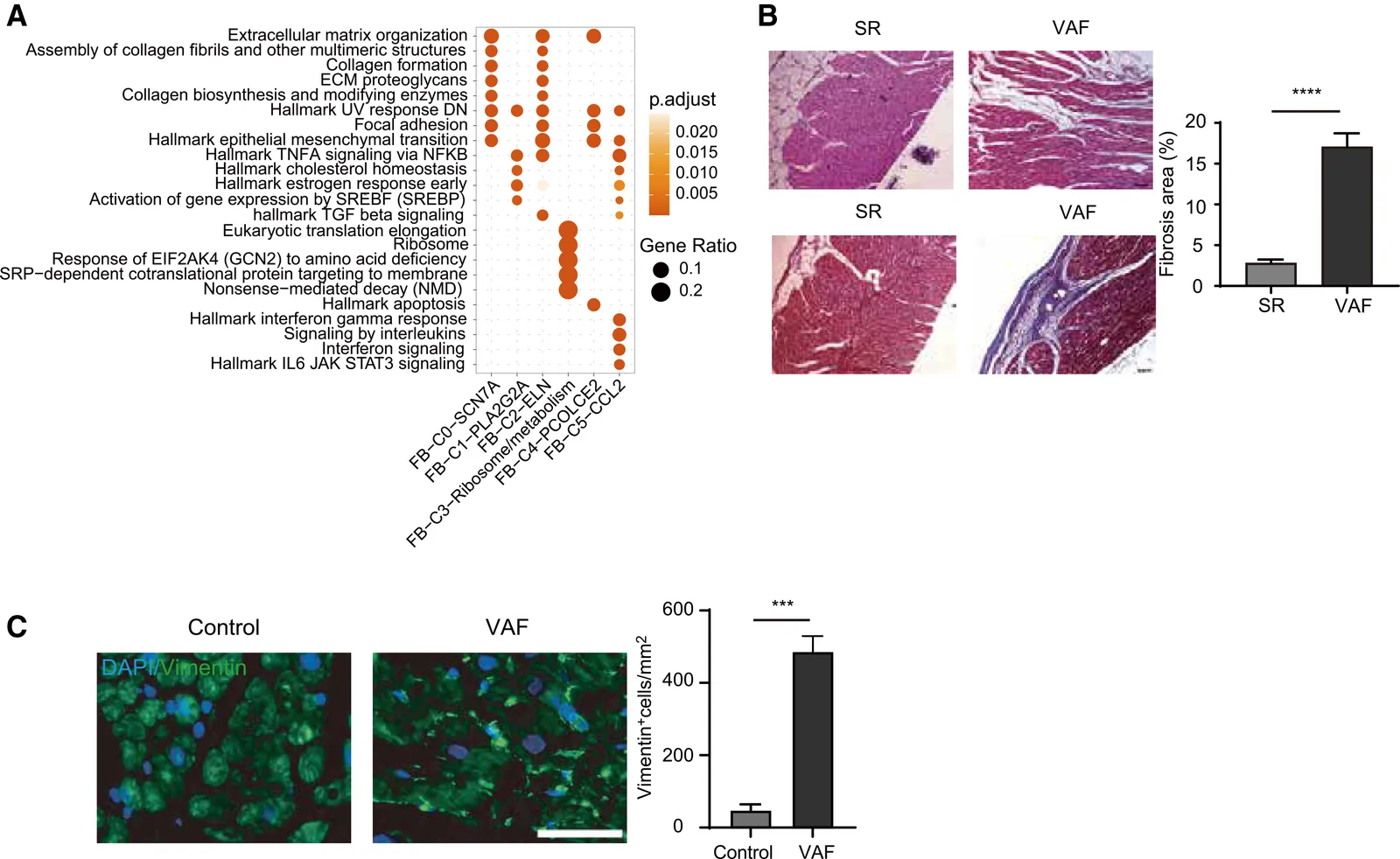
Pathway enrichment in cardiac fibroblast subsets and cardiac fibrosis in VAF hearts (A) Pathways enriched by differentially expressed genes within six fibroblast subsets. GeneRatio represents the ratio of DEGs in a specific pathway to the total number of genes in that pathway. The color indicates the adjusted *p* value by the pathway enrichment analysis. (B) Representative images of H&E and Masson staining of SR and VAF hearts, with quantification of the percentage of cardiac fibrosis in atrium (*n* = 2–8, biological replicates, each consisting of five technical replicates, *p* < 0.0001). Scale bars, 200 μm. Our study integrated data from both healthy and VAF patients. (C) Representative images of immunostaining of vimentin in control or VAF hearts, with quantification of vimentin^+^ fibroblasts. The experiment was conducted with 2–5 biological replicates, each consisting of five technical replicates (*p* < 0.0001). Data were shown as mean ± S.E.M, scale bars, 50 μm; the *t* test was used for comparisons between two groups; *p* < 0.05 was considered as statistical significance in this study. ∗*p* < 0.05, ∗∗*p* < 0.01, ∗∗∗*p* < 0.001, and ∗∗∗∗*p* < 0.0001.

Monocle analysis identified that FB-C3-ribosome/metabolism at lower pseudotime displayed comparatively higher expression of homeostatic fibroblast signatures, whereas FB-C2-ELN at higher pseudotime exerted upregulation of ECM and myofibroblast-associated programs (Figures 2D–2G). In VAF patients, FB-C2-ELN group was at the highest pseudotime, however in SR group, FB-C5-CCL2 was at the highest pseudotime (Figures 2D–2F), suggesting that FB-C2-ELN was closely associated with VAF pathogenesis. All other fibroblasts seemed to exist in a state of high entropy, suggesting substantial plasticity within these populations (Figures 2D–2F). Pathway analysis comparing fibroblast states identified distinct pathway enrichment, including ECM synthesis and assembly enriched in FB-C0-SCN7A and FB-C2-ELN, TGF-β signaling in FB-C1-PLA2G2A, interferon gamma response in FB-C3-ribosome/metabolism, epithelial mesenchymal transition in FB-C4-PCOLCE2, and interleukin signaling in FB-C5-CCL2 (Figure 3A). Our further histological analysis of human hearts validated pathological cardiac fibrosis in VAF hearts (Figures 3B and 3C).

### Differential gene expression analysis reveals the involvement of ITGA8 in VAF-related FB-C2-ELN

**Figure 4:**
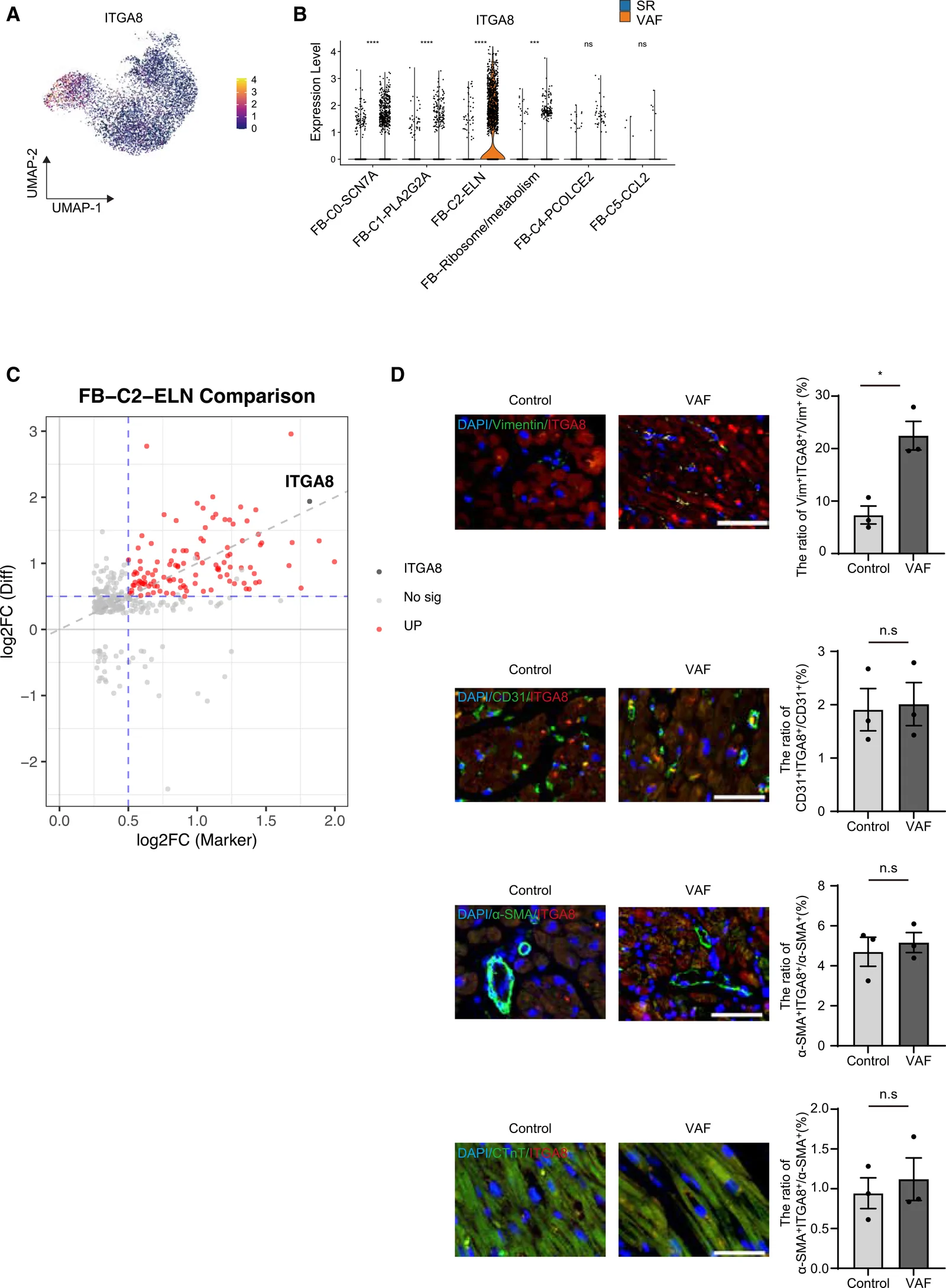
Expression of ITGA8 on cardiac fibroblasts (A) UMAP of fibroblast subclustering and the cardiac fibroblast subsets that express ITGA8. Notably, ITGA8 is specifically expressed in FB-C2-ELN. (B) The differential expression of ITGA8 between SR (blue) and VAF (orange) in six fibroblast subsets. (C) The differential expression between VAF and SR in FB-C2-ELN (*y* axis, log2FC [VAF vs. SR]) and the differential expression between FB-C2-ELN and other fibroblast subsets (*x* axis, log2FC [FB-C2-ELN vs. other fibroblast subsets]). Red points indicated log2FC (VAF vs. SR) > 0.5, log2FC (FB-C2-ELN vs. other fibroblast subsets) > 0.5, adjusted *p* value of both VAF vs. SR and FB-C2-ELN vs. other fibroblast subsets <0.05. Black point indicated ITGA8. Gray point indicated log2FC (VAF vs. SR) < 0.5 or log2FC (FB-C2-ELN vs. other fibroblast subsets) < 0.5 or adjusted *p* value of both VAF vs. SR and FB-C2-ELN vs. other fibroblast subsets >0.05. (D) Representative images of co-immunostaining of vimentin/ITGA8, CD31/ITGA8, α-SMA/ITGA8, and CTnT/ITGA8 in SR or VAF hearts, with quantification of vimentin^+^ITGA8^+^ cells in vimentin^+^ fibroblasts (*n* = 3, *p* = 0.0104), CD31^+^ITGA8^+^ cells in CD31^+^ endothelial cells (*n* = 3, *p* = 0.8639), α-SMA^+^ITGA8^+^ cells in α-SMA^+^ smooth muscle cells (*n* = 3, *p* = 0.6278) and cTnT^+^ITGA8^+^ cells in cTnT^+^ myocardial cells (*n* = 3, *p* = 0.6224). Scale bars, 50 μm. Data were shown as mean ± S.E.M, scale bars, 50 μm, the *t* test was used for comparisons between two groups; *p* < 0.05 was considered as statistical significance in this study. n.s, no significant statistical differences. ∗*p* < 0.05, ∗∗*p* < 0.01, ∗∗∗*p* < 0.001, and ∗∗∗∗*p* < 0.0001.

**Figure 5:**
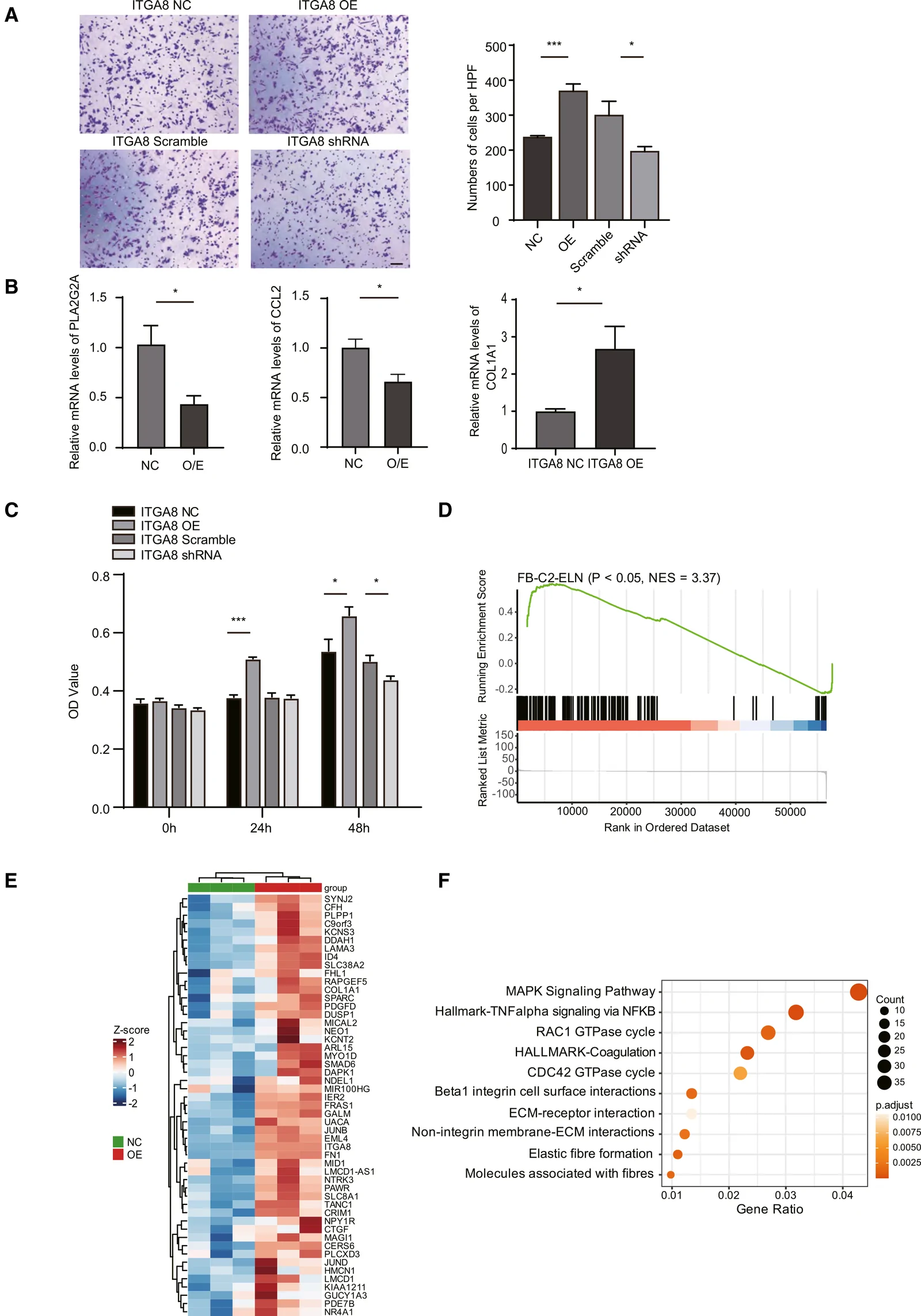
Functional impact of ITGA8 on cardiac fibroblasts and fibrosis (A) ITGA8 promotes the ability of migration of HCFCs, as shown by transwell chemotactic assay (*n* = 5; negative control [NC] vs. OE: *p* = 0.0003; Scramble vs. shRNA: *p* = 0.0438). Scale bars, 200 μm. (B) Relative mRNA expression levels of PLA2G2A (*n* = 3, *p* = 0.0427), CCL2 (*n* = 3, *p* = 0.0340) and COL1A1(*n* = 3, *p* = 0.0481) in ITGA8 NC and ITGA8 O/E cells (*n* = 3). (C) ITGA8 promotes the ability of proliferation of HCFCs, as shown by MTT assay (*n* = 3; ITGA8 NC vs. ITGA8 OE at 24 h: *p* < 0.0001; ITGA8 NC vs. ITGA8 OE at 48 h: *p* = 0.0384; ITGA8 Scramble vs. ITGA8 shRNA at 48 h: *p* = 0.0212). (D) The gene set enrichment analysis (GSEA) shows that the marker genes of FB-C2-ELN are upregulated in fibroblasts with ITGA8 overexpression. (E) The upregulated marker genes of FB-C2-ELN in fibroblasts with ITGA8 overexpression (*p* < 0.05). (F) Pathways enriched by the upregulated genes in fibroblasts with ITGA8 overexpression. GeneRatio represents the ratio of DEGs in a specific pathway to the total number of genes in that pathway. The color indicates the adjusted *p* value by the pathway enrichment analysis. NC, negative controls. O/E, ITGA8 overexpression. Scramble, negative controls. shRNA, ITGA8 gene silencing. Data were shown as mean ± S.E.M. The *t* test was used for comparisons between two groups, n.s, no significant statistical differences. ∗*p* < 0.05; ∗∗*p* < 0.01, ∗∗∗*p* < 0.001, and ∗∗∗∗*p* < 0.0001. Our study integrated data from both healthy and VAF patients.

Notably, we identified ITGA8 enriched in cardiac fibroblasts, especially in FB-C2-ELN subpopulation, which might be involved in the regulation of cardiac fibrosis (Figures 4A and 4B). To clarify the basis for prioritizing ITGA8, we detail our analytical workflow as follows: after identifying the expanded FB-C2-ELN fibroblast subcluster in VAF, we performed marker analysis (FB-C2-ELN versus other fibroblasts) followed by differential expression analysis (VAF versus donor) specifically within this subcluster. Integration of these results highlighted ITGA8 as the top candidate (Figure 4C). Co-immunostaining of ITGA8 and vimentin (a typical maker for all cardiac fibroblasts) showed the specific fibroblast subpopulation vimentin^+^ITGA8^+^ (Figure 4D) represented as FB-C2-ELN detected by snRNA-seq (Figure 4B). Moreover, we observed higher number of vimentin^+^ITGA8^+^ fibroblasts in VAF patients compared with SR (Figure 4D), suggesting that this specific fibroblast subpopulation essentially contributed to VAF-induced cardiac fibrosis, providing a potential therapeutic target against cardiac fibrosis. However, there were no differences in CD31^+^ITGA8^+^ (CD31, EC marker), CTnT^+^ITGA8^+^ (CTnT, CM marker), and α-SMA^+^ITGA8^+^ (α-SMA, smooth muscle cell marker) between VAF patients and control group (Figure 4D), suggesting the expression of ITGA8 in ECs, CMs, and smooth muscle cells may not significantly contribute to the pathology of VAF. Our further *in vitro* study showed that ITGA8 overexpression enhanced cardiac fibroblast proliferation and migration, as well as in enhancing ECM synthesis by these cells, whereas ITGA8 knockdown reduced fibroblast proliferation and migration(Figures 5A–5C), suggesting that ITGA8 is involved in the regulation of cardiac fibrosis. RNA sequencing of ITGA8-overexpressing fibroblasts showed that the marker genes of FB-C2-ELN were significantly upregulated in fibroblasts with ITGA8 overexpression (Figures 5D and 5E). Our *in vitro* data showed that ITGA8 overexpression significantly reduced the expression of CCL2 and PLA2G2A mRNA levels (Figure 5B), suggesting that ITGA8 expression negatively correlates with the expression of CCL2 and PLA2G2A in FB-C1 and FB-C5 subsets, respectively (Figure 4B). Particularly, the upregulated genes in fibroblasts with ITGA8 overexpression were enriched in TNF alpha signaling, ECM-receptor interaction, and epithelial mesenchymal transition, which play key roles in the regulations of cardiac fibrosis (Figures 5E and 5F).

### Subclustering of monocytes/macrophages and subsequent analysis of tissue repair-related gene expression

**Figure 6:**
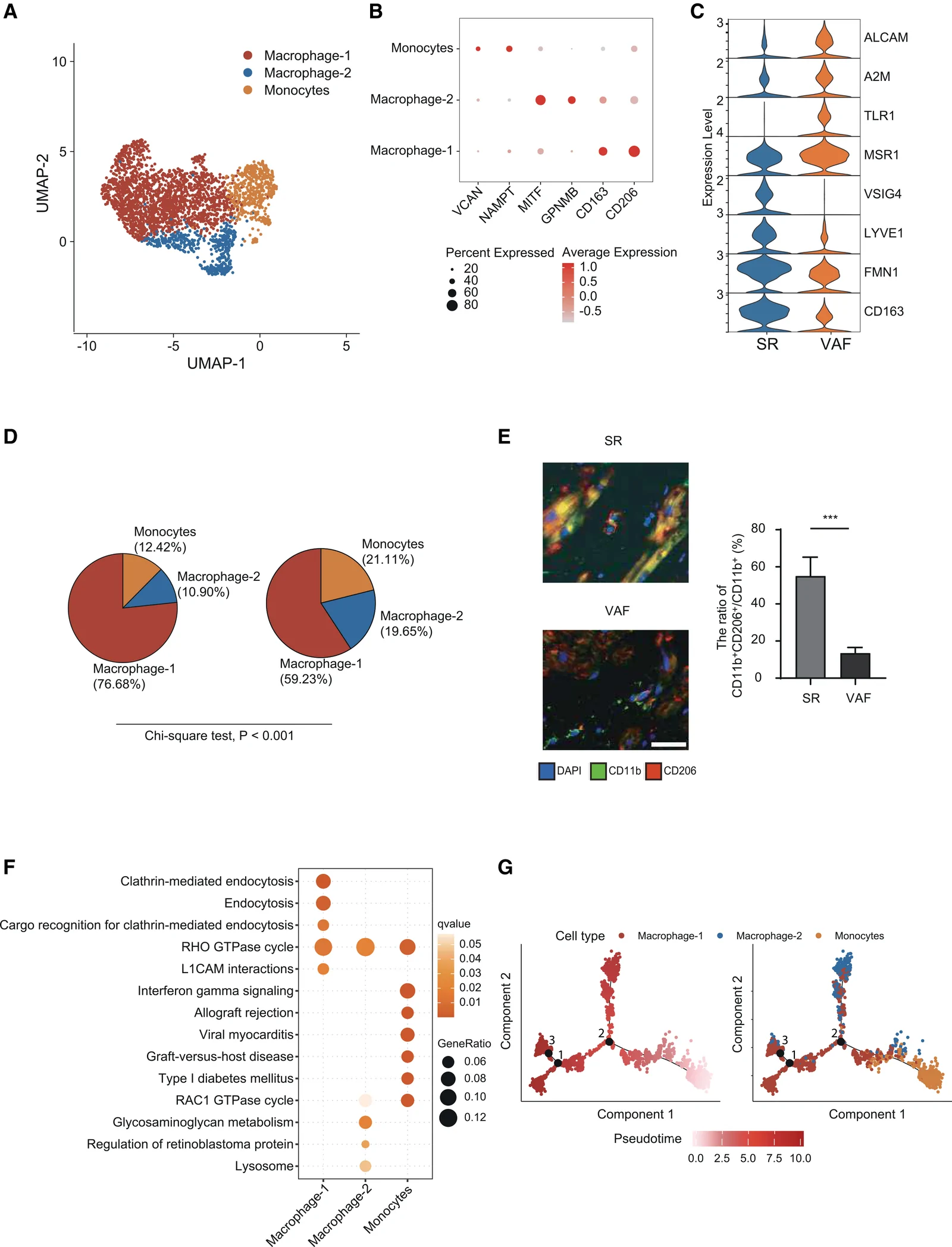
Subclustering of monocytes/macrophages and subsequent analysis of tissue repair-related gene expression (A) The three distinct monocyte/macrophage subsets identified by the subclustering of monocytes/macrophages. As for monocytes/macrophages, we first subset myeloid cells, then we re-normalized, re-scaled, re-PCA, and re-clustered. Then, we used VCAN, NAMPT, MITF, GPNMB, CD163, and MRC1 as markers for subclustering. (B) The signature genes highly expressed in monocytes/macrophages subsets. The color represents the average expression and the point size represents the percentage of cells expressing the marker genes. (C) The representative signatures differentially expressed between SR (blue) and VAF (orange) in monocyte/macrophage. (D) The proportion of three monocytes/macrophages subsets in SR (left) and VAF (right). Data was analyzed by the chi-square test. (E) Representative images of co-immunostaining of CD11b/CD206 in SR or VAF hearts, with quantification of CD11b^+^CD206^+^ cells in CD11b^+^ micrographs. The experiment was conducted with 2–5 biological replicates, each consisting of five technical replicates (*p* < 0.0001). Data were shown as mean ± S.E.M, scale bars, 50 μm. The *t* test was used for comparisons between two groups, ∗∗∗*p* < 0.001. (F) Pathways enriched by differentially expressed genes in three monocytes/macrophage subsets. GeneRatio represents the ratio of DEGs in a specific pathway to the total number of genes in that pathway. The color indicates the adjusted *p* value by the pathway enrichment analysis. (G) Monocle2 trajectory analysis of monocytes/macrophages colored by pseudotime (left) and monocytes/macrophages subsets (right). Scale bars, 50 μm; ∗*p* < 0.05; ∗∗*p* < 0.01, ∗∗∗*p* < 0.001, and ∗∗∗∗*p* < 0.0001. Our study integrated data from both healthy and VAF patients.

Unsupervised clustering analysis revealed large populations of macrophages and dendritic cells in VAF and SR patients (Figure 6A). Our snRNA-seq data showed two distinct macrophage population: macrophage-1 (CD163 and CD206), macrophage-2 (MITF and GPNMB), and monocytes (VCAN and NAMPT) (Figures 6A and 6B). Differential expression analysis by single-nuclear approaches across disease states revealed a large number of genes significantly upregulated (ALCAM, A2M, TLR1, and MSR1) and downregulated (VSIG4, LYVE1, FMN1, and CD163) in VAF samples compared with SR (Figure 6C). VAF samples displayed reduced numbers of CD11b^+^/CD206^+^ macrophages, macrophage-1, and a greater number of macrophage-2 and monocytes (Figure 6D). We further performed co-immunostaining of CD11b and CD206 in heart tissues and confirmed that CD11b^+^CD206^+^ macrophages decreased in VAF samples compared with SR (Figure 6E). Pathway analysis identified upregulation of multiple pathways in VAF samples including interferon gamma signaling, allograft rejection, and viral myocarditis signaling pathway in monocyte subset, and Rho GTPase activity and lysosome signaling pathways in macrophage-2 subset (Figure 6F).

We utilized Monocle to calculate the pseudotime for the monocytes/macrophages. Our data showed that monocyte exerted the characteristic of the most progenitor-like state. In comparison with SR, VAF hearts displayed a larger abundance of macrophage-1 subsets with intermediate transcriptional continuum along the macrophage trajectory. Superimposing cluster identities showed that these cells belonged to the monocyte cluster, suggesting that they are monocyte-derived (Figure 6G). These data demonstrate that a link between monocyte-derived macrophages and inflammation in VAF.

### Subclustering of CMs and ECs

Our differential expression analysis of CMs by single-nucleus approaches was performed and revealed that 788 genes were upregulated ranked by log2 fold-change comparing SR with VAF, including many upregulated genes (natriuretic peptides B [NPPB], ANKRD1, TCAP, MB, ACTC etc.). Four subpopulations of CMs with differing gene expression signatures were identified by unsupervised clustering (Figure S2A). Both CMs from SR and VAF samples existed in all 4 subpopulations (Figures S2B, S3A, and S3B) characterized by specific gene expression (Figure S2C). However, CMs from VAF samples showed a higher prevalence of CM1 and CM2 markers (e.g., NPPB/Matrix Gla protein [MGP]/MYH7 and PALLD/RNF150/PXDNL, respectively), while simultaneously showing a significant reduction in those expressing markers for CM3 and CM4 (e.g., MB/COX6A2/COX7A1 and KCND3/LIMCH1/PALMD/RYR2, respectively). Pathway enrichment analyses performed on each state of CM cell identified distinct pathways, including hypertrophic CM in CM1, calcium signaling in CM2, oxidative phosphorylation in CM3, and cardiac muscle contraction in CM4 (Figure S2D). In addition, we observed a global increase in MGP and NPPB expression in VAF (Figure S2E). Furthermore, using entropy versus pseudotime analysis for each CM cluster, we disclosed that SR hearts contained higher cell abundance with more transcriptional states to express high RYR2 than VAF hearts (Figures S3C and S3D). Our observations demonstrated a convergence toward disease-associated CM phenotypes in VAF.

We next identified the following distinct endocardial subtypes: capillary EC (CapEC and RGCC), artery EC (ArtEC and SEMA3G), and veinous EC (VEC, PLVAP, and ACKR1) (Figures S4A and S4B). As shown in Figure S4C, the three EC subsets had distinct gene expression profiles. Kyoto Encyclopedia of Genes and Genomes (KEGG) pathway analysis revealed that TGF-β, focal adhesion, and platelet activation signaling pathways were enriched in ECs obtained from VAF compared with SR samples (Figure S4E), whereas adhesion junction, sphingosine metabolism, and Jak-STAT signaling pathways displayed enrichment in ECs from SR hearts. It has been recently reported that cardiac ECs underwent endothelial-to-mesenchymal activation (EndMA) during the process of cardiac fibrosis after myocardial infarction.^19^ Interestingly, our investigation revealed multiple mesenchymal signatures associated with VAF disease state. VIM, FN1, and DCN were upregulated in VAF ECs, while EC markers, including PECAM, KDR and FLT1, were downregulated in VAF ECs, suggesting that cardiac ECs might engage in EndMA during the process of VAF-related cardiac fibrosis (Figure S4F). Collectively, our data suggested that cardiac ECs reduced their abundance and displayed EndMA in VAF, providing new insights in the pathogenesis of human VAF.

### Aberrant cross-signaling between cells in VAF heart

**Figure 7:**
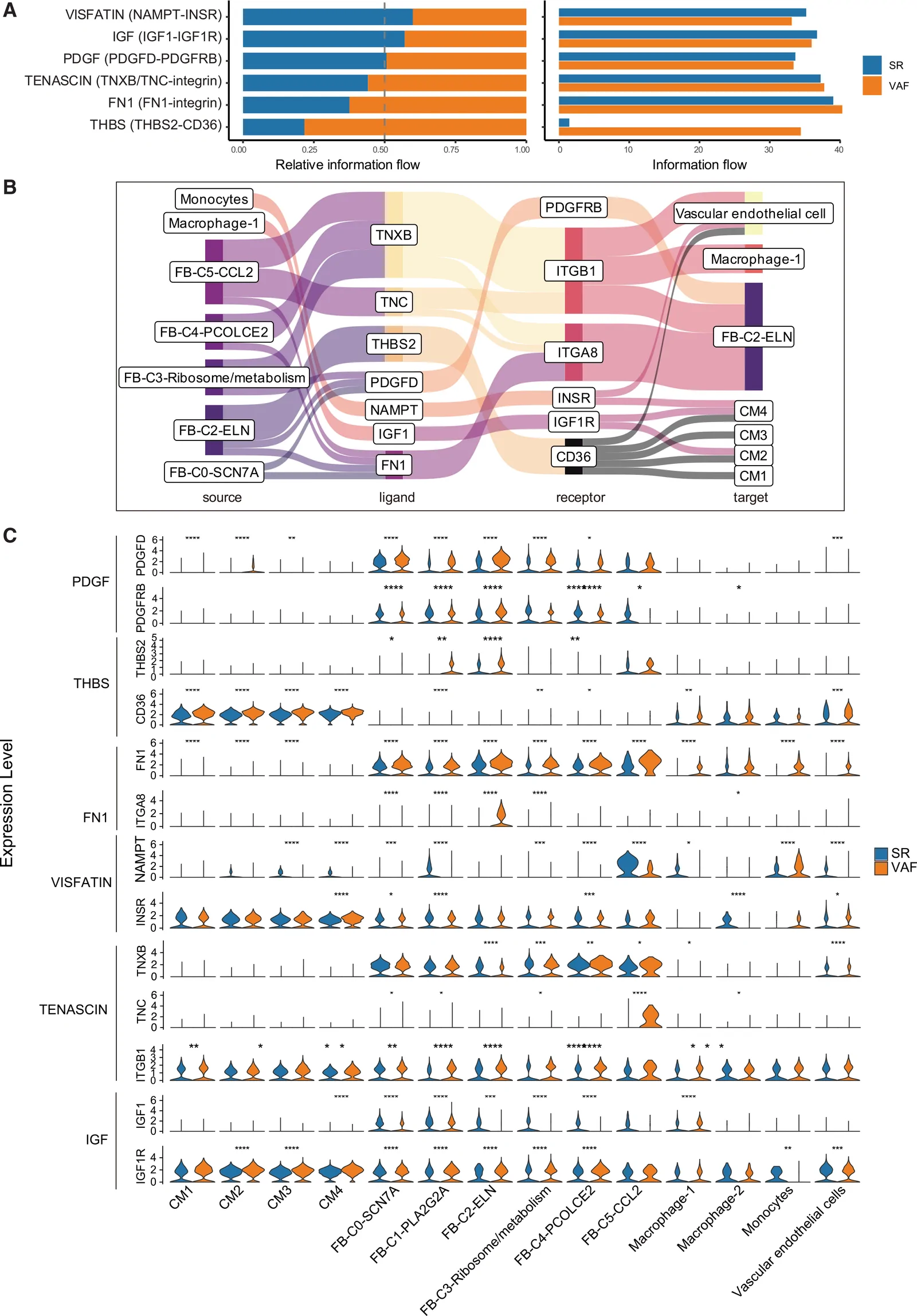
The cell-cell communication between VAF-related cell types (A) The overall information flow of signaling pathways with differentially expressed ligand-receptor pairs between VAF and SR. The ligand-receptor pairs should be jointly upregulated or downregulated in certain cell type of VAF (Wilcoxon rank-sum test, *p* < 0.05). (B) The cell-cell communication network constructed by the VAF-related cell types and the corresponding ligands and receptors highly expressed by those cell types. The ligands and receptors were selected based on their higher expression in VAF compared to SR (adjusted *p* < 0.05). (C) The differential expression levels of genes encoding the ligands and receptors within the cell subsets between VAF and SR. The *t* test was used for comparisons between two groups; n.s, no significant statistical differences. ∗*p* < 0.05; ∗∗*p* < 0.01, ∗∗∗*p* < 0.001, and ∗∗∗∗*p* < 0.0001. Our study integrated data from both healthy and VAF patients.

Comparative analysis of cell-cell communication between VAF and SR samples using CellChat identified six signaling pathways with differentially expressed ligand-receptor pairs. Among these, the THBS and FN1 pathways exhibited significantly higher information flow in VAF, suggesting their potential critical roles in VAF pathogenesis (Figure 7A). Ligand-receptor pairs in THBS, FN1, TENASCIN, PDGF, and VISFATIN pathways were upregulated in VAF (Figures 7B and 7C).

To determine the cellular source driving these pathway alterations, we focused on the FB-C2-ELN fibroblast subcluster—a population identified by per-patient proportion analysis as significantly expanded in VAF. We therefore hypothesized it acts as a critical signaling hub. Our focused analysis validated this, revealing that three ligands specifically upregulated within FB-C2-ELN—THBS2, PDGFD, and FN1—served as major signal sources. They engaged receptors such as CD36, PDGFRB, and integrins on recipient cells, thereby conveying enhanced outgoing communication to key cellular targets including vascular ECs, other FB-C2-ELN fibroblasts, and CMs (Figures 7B and 7C). This pattern confirms that the THBS and FN1 pathways, with elevated information flow in VAF, are central to the aberrant cross-signaling mediated by this expanded fibroblast population.

Consistent with our prior findings, CellChat analysis further showed that ITGA8 was significantly upregulated in the FB-C2-ELN population, underscoring the likely importance of fibroblast-expressed ITGA8 in VAF pathogenesis (Figures 7B and 7C).

### ITGA8 knockdown reduces AF susceptibility and ameliorates atrial fibrosis in the ACh-CaCl2-induced rat model

**Figure 8:**
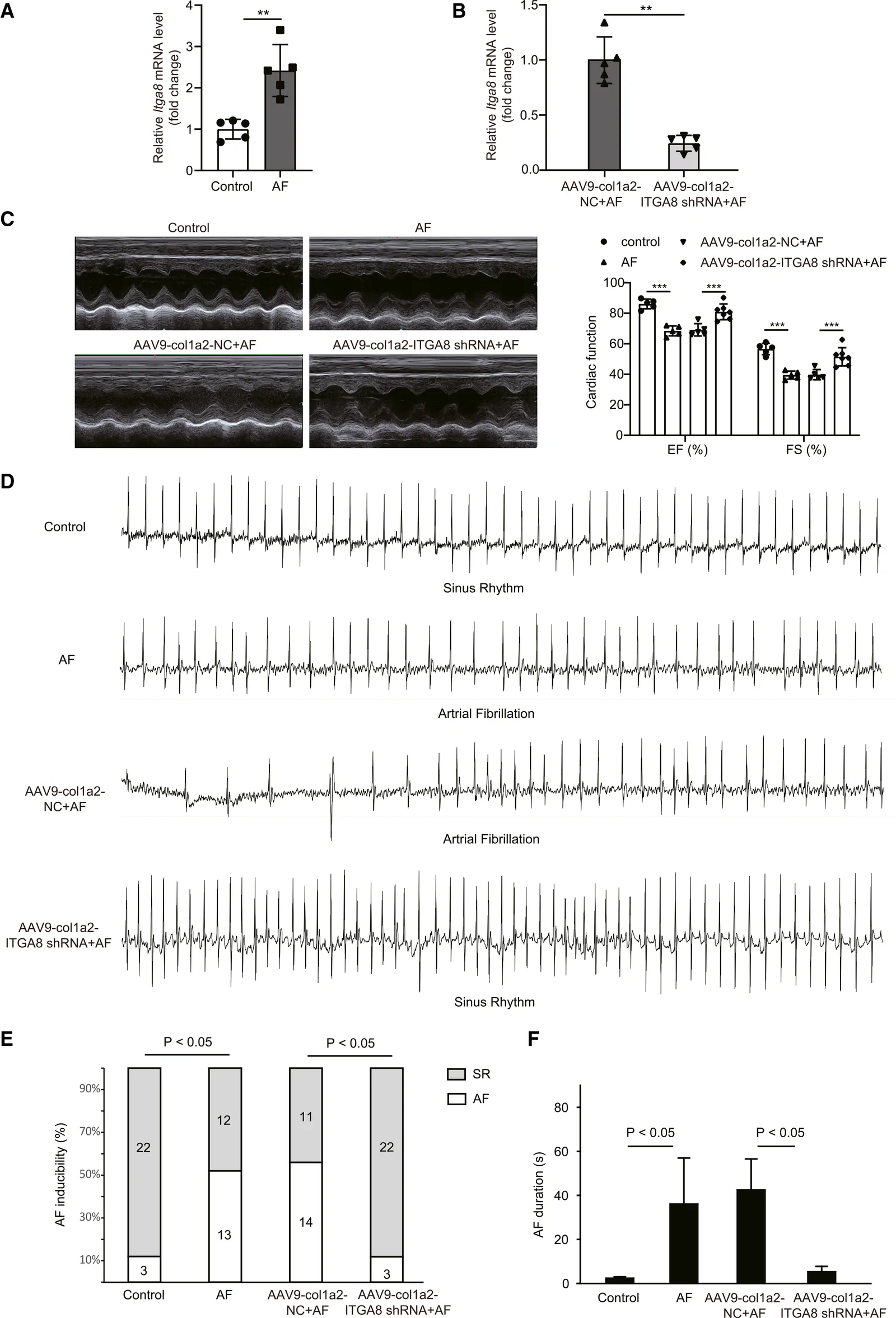
AAV9-col1a2-ITGA8 knockdown reduces AF susceptibility in rats (A) The relative expression levels of Itga8 mRNA in rat atriums in the control and AF groups (*n* = 5, *p* = 0.0079). (B) The relative expression levels of Itga8 mRNA in rat atriums in the AAV9-col1a2-NC and AAV9-col1a2-ITGA8 shRNA groups (*n* = 5, *p* = 0.0079). (C) Representative M-mode echocardiographic images from the indicated groups, with quantification of left ventricular ejection fraction (EF) and fractional shortening (FS), showing reduced cardiac function in AF and AAV9-col1a2-NC+AF rats and improved in the AAV9-col1a2-ITGA8 shRNA group (n = 5–7, control vs. AF for EF: *p* < 0.0001, AAV9-col1a2-NC+AF vs. AAV9-col1a2-ITGA8 shRNA+AF for EF: *p* = 0.0002, Control vs. AF for FS: *p* < 0.0001, AAV9-col1a2-NC+AF vs. AAV9-col1a2-ITGA8 shRNA+AF for FS: *p* = 0.0004). (D) Representative ECG traces showing sinus rhythm or AF in each group. (E) AF inducibility in control, AF, AAV9-col1a2-NC+AF, and AAV9-col1a2-ITGA8 shRNA+AF groups (5 bursts per animal, 5 animals per group, control vs. AF: *p* = 0.0054, AAV9-col1a2-NC+AF vs. AAV9-col1a2-ITGA8 shRNA+AF: *p* = 0.0023). (F) AF duration after burst pacing in each group (5 bursts per animal, 5 animals per group, control vs. AF: *p* = 0.0362, AAV9-col1a2-NC+AF vs. AAV9-col1a2-ITGA8 shRNA+AF: *p* = 0.0491). Data are shown as mean ± S.E.M. The chi-square test was used for AF inducibility, and one-way ANOVA was used for continuous variables. *p* < 0.05 was considered statistically significant. ∗∗*p* < 0.01 and ∗∗∗*p* < 0.001.

**Figure 9:**
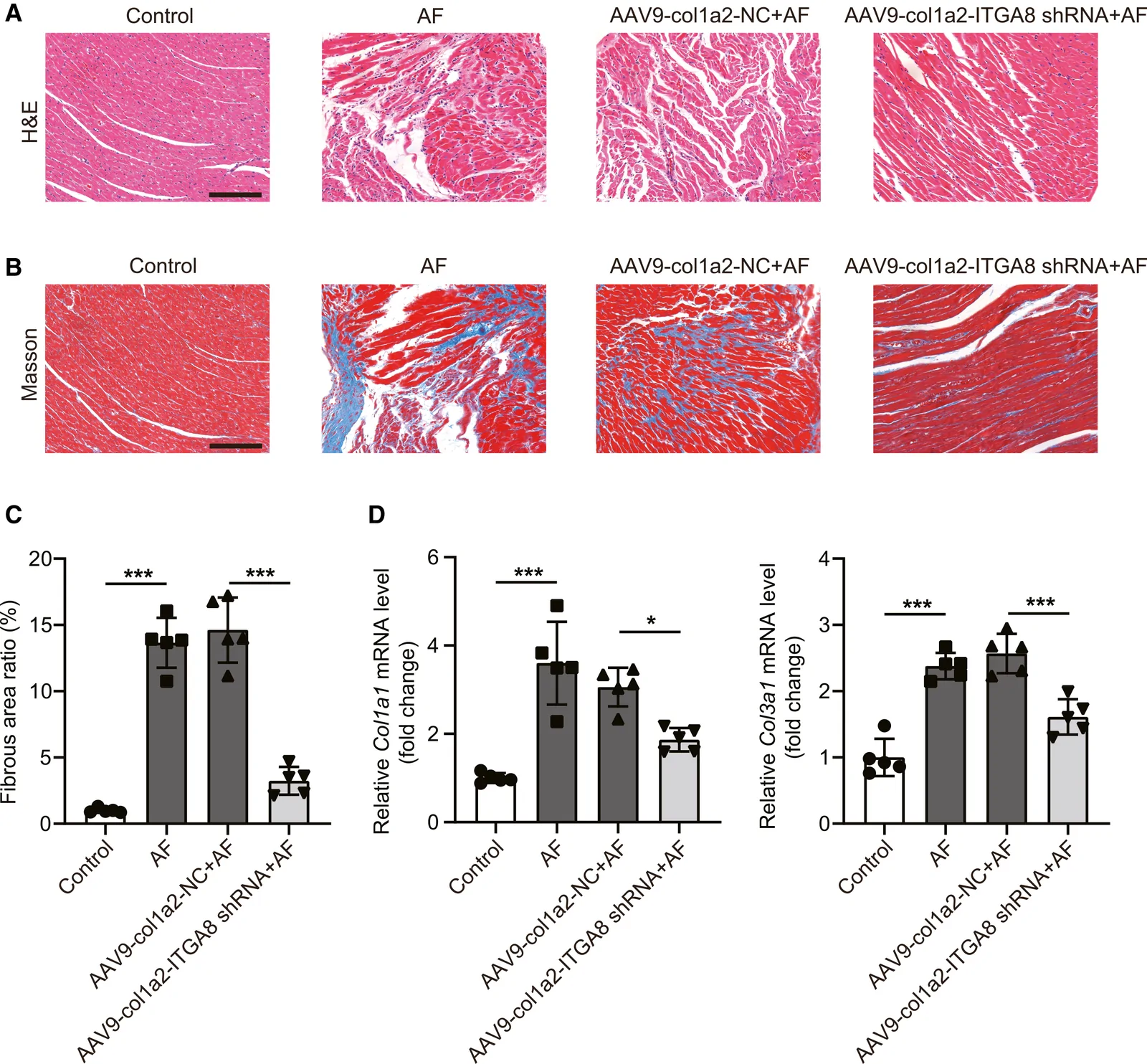
AAV9-col1a2-ITGA8 knockdown reduces atrial fibrosis in rat AF models (A) Representative H&E staining images of rat atrium in the indicated groups. (B and C) Representative Masson staining images of rat atrium in the indicated groups (B), with the quantification of atrial fibrosis (C) (*n* = 5, control vs. AF: *p* < 0.0001, AAV9-col1a2-NC+AF vs. AAV9-col1a2-ITGA8 shRNA+AF: *p* < 0.0001). (D) Relative mRNA expression levels of fibrosis-related genes, including Col1a1 and Col3a1, in rat atrium in the indicted group (*n* = 5, control vs. AF for Col1a1: *p* < 0.0001, AAV9-col1a2-NC+AF vs. AAV9-col1a2-ITGA8 shRNA+AF for Col1a1: *p* = 0.0137, control vs. AF for Col3a1: *p* < 0.0001, and AAV9-col1a2-NC+AF vs. AAV9-col1a2-ITGA8 shRNA+AF for Col3a1: *p* = 0.0002). Data are shown as mean ± S.E.M. Scale bars, 50 μm. One-way ANOVA was used for continuous variables. *p* < 0.05 was considered statistically significant. ∗*p* < 0.05 and ∗∗∗*p* < 0.001.

To further validate the functional relevance of the ITGA8-associated fibroblast program *in vivo*, we performed an AAV-mediated small hairpin RNA (shRNA) knockdown of ITGA8 in rats subjected to ACh/CaCl2-induced AF. In the established AF rats, we observed significant upregulation of *Itga8* expression in AF rat atrium, compared to sham controls (Figure 8A). Efficient knockdown of *Itga8* in the rat atrium was confirmed in the AAV9-col1a2-ITGA8 shRNA group (Figure 8B). Echocardiographic analysis showed that left ventricular ejection fraction (EF) and fractional shortening (FS)were markedly reduced in the AF and AAV9-col1a2-NC+AF groups compared with the control group, whereas the AAV9-col1a2-ITGA8 shRNA+AF group exhibited improved EF and FS (Figures 8B and 8C). Importantly, ACh/CaCl2-induced AF modeling markedly increased AF inducibility and prolonged AF duration, compared to the control group (Figures 8D–8F). In contrast, AAV9-col1a2-ITGA8 shRNA significantly reduced AF inducibility and shortened AF duration compared with the AAV9-col1a2-NC+AF groups (Figures 8D–8F). Histological analysis further demonstrated pronounced atrial fibrosis in the AF group, compared with the control group, and the increased fibrosis was substantially attenuated in the AAV9-col1a2-ITGA8 shRNA group (Figures 9A–9C). Accordingly, fibrosis-related genes were sharply reduced in the AAV9-col1a2-ITGA8 shRNA group (Figure 9D). Collectively, these findings provide *in vivo* evidence that ITGA8 contributes to AF susceptibility and atrial fibrotic remodeling, and ITGA8 knockdown mitigates AF vulnerability and pathological atrial remodeling.

## Discussion

scRNA-seq technology has emerged as a powerful tool in biomedical research, which enables us to deeply disclose the characterization of cell subpopulations in healthy and diseased human organs. In the field of CAD study, this technique has been increasingly employed to uncover CAD-associated alteration in cell subpopulation and their transcriptome. In our study, we utilized single-nucleus sequencing to dissect atrial cardiac cells in healthy and VAF individuals. Twelve major cardiac cell types from 37,425 nuclei collected from the left atrium of VAF and SR samples were identified. We observed the distinct gene expression profile in different cell population, with a significant association with VAF. The application of this technology enabled us to comprehensively dissect the gene expression profile of individual cardiac nucleus and thereby identify disease-specific changes in gene expression patterns in VAF. Our findings may help us better understand the molecular mechanisms underlying VAF and could have implications for the development of novel therapeutic approaches to manage this common cardiac arrhythmia.

Our investigation study revealed that there are significant differences in the transcriptome of CMs between patients with VAF and SR. We identified 4 distinct subpopulations of CMs, and our data revealed that patients with VAF exhibit an upregulation of NPPB/MGP expression in subpopulations. These findings align with and expand upon the current understanding of VAF pathogenesis, highlighting the importance of CM-specific gene regulation in VAF. It has long been known that NPPB, which encodes for B-type natriuretic peptide (BNP) is a biomarker for heart failure.^20^ Previous clinical studies showed that the increased levels of BNP are a predictive factor of incidental AF.^21^ These findings suggest that BNP may play a role in the development and progression of AF.^21^ In consistence with these reports, we identified NPPB is upregulated in CM subsets from atrium of VAF patients, indicating that NPPB may have potential as biomarkers for VAF. MGP is another intriguing target identified in our study. It has been shown that MGP is involved in the prevention of arterial calcification, which is an independent predictor of cardiovascular morbidity and mortality.^22^ Although MGP’s role in VAF is not fully understood, recent evidence suggests that vascular calcification and arterial stiffness are closely associated with AF development.^23^^,^^24^ Our results showed that the elevated MGP expression in CM subpopulation may play a role in VAF-associated remodeling, potentially influencing arterial stiffness and atrial structural changes that promote arrhythmogenesis.^23^^,^^24^ Given the known association between VAF and arterial stiffness, further investigation into the role of MGP in VAF pathophysiology is warranted.

Present study revealed a reduction in the overall count of ECs in patients with AF,^25^ which was further confirmed by our histological observations. We further identified three major subpopulations of ECs, including EC, ArtEC, and VEC, by snRNA-seq. Our results showed significant alterations in the expression profiles of EC subpopulations between VAF patients and SR. Notably, our analysis revealed multiple mesenchymal signatures enriched in cardiac ECs from VAF patients. In particular, VIM, FN1, and DCN were upregulated in VAF ECs, while EC markers such as PECAM, KDR, and FLT1 was downregulated in VAF ECs, suggesting that cardiac ECs might undergo EndMA in VAF hearts.^19^ These changes suggest that cardiac ECs in VAF undergo EndMA, a process that has been implicated in pathological cardiac remodeling.^26^ EndMA is known to be induced by hypoxic and inflammatory environments, which are prevalent in VAF, and it contributes to the loss of endothelial characteristics while promoting a mesenchymal, fibrotic phenotype.^27^ It has been reported that EndMA was involved in the regulation of pathological cardiac fibrosis.^19^ Our study indicates that EndMA also exists in the process of VAF-induced cardiac remodeling, which potentially provides a novel therapeutic target for VAF.

To disclose the expression characteristics of cardiac fibroblasts in VAF hearts, the expression profile differential analysis was employed in fibroblasts from patients with VAF. Our findings revealed that fibroblasts from patients with VAF displayed a significant enrichment of ECM-related genes. The histological examination of cardiac tissue samples obtained from VAF patients corroborated these RNA sequencing results, showing a marked increase in cardiac fibrosis. It has been showed that cardiac fibroblasts displayed a high degree of cell heterogeneity.^8^^,^^28^^,^^29^^,^^30^ In line with these findings, our study identified six distinct fibroblast cell subpopulations, including FB-C0, FB-C1, FB-C2, FB-C3, FB-C4, and FB-C5. Importantly, a significant enrichment of FB-C2-ELN fibroblast cells was observed within the VAF patient group. These results agree with a recent publication in a recent report.^7^ Additionally, the pseudo time analysis indicated that fibroblast cells from the VAF group exhibited coordinated upregulation of ECM and myofibroblast-associated programs. A previous study on tumor microenvironment showed that ELN-positive myofibroblast was a subtype of myofibroblasts that was nearly absent in normal tissues, and substantially more abundant in oral squamous cell carcinoma (OSCC) tissues.^31^ They reported that ELN-positive myofibroblast was involved in the malignant transformation of squamous cell carcinoma and promotes an immunosuppressive microenvironment by attracting and retaining Tregs.^31^ A recent study on human heart tissues showed that a heart failure-related fibroblast subpopulations characterized by the expression of ELN, which represented as the most differentiated cell states based on low entropy and high pseudo-time values.^7^ In consistence with these reports, we observed that VAF-specific ELN positive fibroblast subpopulation, FB-C2-ELN, suggesting that ELN positive fibroblast might play an important role of organ fibrosis. Most importantly, our data showed that ITGA8 was identified as a prioritized candidate of cardiac fibrosis associated with VAF. The histological analysis demonstrated ITGA8-positive fibroblasts significantly increased in patients with VAF, highlighting the potential utility of ITGA8 as a diagnostic biomarker for assessing pathological changes in human cardiac tissue. Previous investigations showed that ITGA8 was involved in the regualtion of fibroblast proliferation and apoptosis in renal glomerular cells.^32^ In a study, ITGA8 was upregulated in specimens from patients with liver fibrosis, indicating the relevance of the findings to human liver fibrosis.^32^ In another study, it was found that ITGA8 expression is especially enriched in interstitial stromal cells following bleomycin-induced fibrosis in the lung, indicating that stromal cell-expressing ITGA8 regulated the process of lung fibrosis, suggesting its broad role across various fibrotic diseases. Interestingly, deletion of Smad2/3, one of the most important regulators in tissue fibrosis, from cardiac fibroblasts during pressure overload-induced cardiac remodeling downregulated the expression of ITGA8, which suggested that ITGA8 might be involved in the regulation of cardiac fibrosis and ECM remodeling.^32^ These findings are consistent with our current results, which demonstrate that ITGA8 enhances fibroblast proliferation, differentiation, migration, and ECM upregulation, indicating its important role in cardiac fibrosis in patients with VAF. By integrating previous findings with our results, it is evident that ITGA8 regulates fibroblast function across multiple organ systems, thereby contributing to pathological fibrosis, including cardiac fibrosis in VAF. Importantly, our findings suggest that ITGA8 may serve as a potential therapeutic target for modulating fibroblast activity and addressing pathological fibrosis associated with VAF. Additionally, recent investigations revealed that ITGA8 was found with robust expression near the inner wall of Schlemm’s canal (SC) endothelium and that ITGA8-positive cells were identified with the properties as SC ECs.^33^ The expression and the potential effects of ITGA8 on cardiac ECs are very interesting and deserve to be further investigated. Although ITGA8 was enriched in activated fibroblast subsets in our atrial snRNA-seq dataset, prior work in kidney injury models indicates that Itga8 signaling can attenuate tubulointerstitial fibrosis and that loss of Itga8 exacerbates renal fibrosis, potentially through modulation of TGF-β signaling and fibroblast activation.^34^ These observations suggest that ITGA8 functions may be context-dependent across organs and injury paradigms.

The ACh-CaCl2 model has been used previously to induce AF susceptibility and atrial remodeling in rats.^15^^,^^16^^,^^17^^,^^18^ To evaluate the functional role of the ITGA8-related remodeling program in this model. ITGA8 knockdown markedly reduced AF inducibility and decreased atrial fibrosis. These phenotypic effects extend our snRNA-seq and *in vitro* observations by linking ITGA8-expressing fibroblasts to AF vulnerability and pathological atrial remodeling *in vivo*. . Taken it together, our study reveals novel findings on the fibroblast composition and distinct expression profiles of patients with VAF, indicating a significant role of fibroblasts in the pathophysiology of VAF. Although we identify and functionally interrogate an ITGA8-enriched fibroblast state, additional studies will be needed to fully define downstream signaling and to establish whether targeting ITGA8 modifies VAF burden in long-term or large-animal models.

The heterogeneity of monocytes/macrophages cells is known as a key regulator in various cardiac pathologies, including ischemic heart diseases, cardiomyopathy, arrhythmias-related cardiac remodeling, and heart failure.^35^ Macrophages, as multifunctional immune cells, play an essential role in the inflammatory response and tissue repair, with their phenotype and function being highly context dependent.^35^ In line with other sc-RNA sequencing studies on human hearts, our study identified three major monocytes/macrophage populations in VAF samples, including monocytes, macrophage-1, and macrophage-2. Of note, the number of macrophage subpopulation was significantly decreased in the heart tissue from VAF patients, suggesting a potential disruption in the homeostatic and reparative mechanisms within the atrial myocardium of VAF patients.

In AF, CCR2^+^Trem2^+^ inflammatory macrophages have been shown to promote the progression of fibrosis and AF by secreting SPP1, implying that a reduction of macrophage subpopulation may contribute to AF.^36^ Indeed, our study revealed a significant reduction in CD11b^+^/CD206^+^ macrophages in VAF patient. In the context of VAF, the reduction of CD11b^+^/CD206^+^ macrophage subpopulation may impair the resolution of inflammation and promote a pro-arrhythmic substrate through inflammatory signaling and fibrotic remodeling. Understanding the balance and function of these subsets in VAF could unveil new therapeutic targets. For instance, strategies aimed at restoring or enhancing the function of protective macrophages may ameliorate VAF-related adverse remodeling.

### Limitations of the study

This study has some limitations. Firstly, patients with VAF were from a singular cohort, potentially overlooking the potential influence of distinct etiologies on cellular heterogeneity and transcriptional profiles. Moreover, the scope of our dataset was confined to transcriptomic information, precluding a comprehensive assessment of cellular dynamics. The incorporation of complementary datasets, encompassing cell surface protein expression and chromatin accessibility, may confer greater granularity to the interpretation of our findings.

Several limitations pertaining to our trajectory inferences must be acknowledged. Pseudotime methods (including Monocle) infer a low-dimensional ordering of transcriptional similarity and, in cross-sectional human specimens, cannot establish true temporal dynamics, lineage relationships, or causality. Accordingly, our pseudotime results are presented as hypothesis-generating descriptions of fibroblast and macrophage state continua that merit validation in longitudinal sampling, perturbation studies, or lineage-traced experimental models.

Despite these limitations, our study represents a comprehensive investigation of the transcriptomic and cellular profile of healthy and VAF hearts. Our analyses shed light on changes in cardiac cell populations during the process of VAF, with disease-specific cellular states emerging as a distinct characteristic. Our data will represent a valuable resource for further investigations, with potential opportunities for therapeutic innovation and development.

## Resource availability

### Lead contact

Further information and requests for resources should be directed to and will be fulfilled by the lead contact, Yangyang Zhang (zhangyangyang_wy@vip.sina.com).

### Materials availability

No unique reagents were generated in this study. Reagents and materials used in this study are commercially available or can be obtained from the corresponding authors upon reasonable request.

### Data and code availability

The raw single-nucleus RNA-sequencing files supporting this study have been deposited in NODE under accession no. OEP00006765. Because these human transcriptomic data are subject to applicable human genetic resources regulations of the People’s Republic of China, the files are available through controlled access rather than unrestricted public download. Qualified researchers may submit an access request through NODE. Requests will be reviewed, and approved applicants will receive access in accordance with applicable legal, ethical, repository, and institutional requirements. This controlled-access arrangement is active and is not an embargo or delayed-release plan. Custom analysis scripts are available at GitHub: https://github.com/YuliangWang316/AF_SinglecellRNAseq and Zenodo (doi: https://doi.org/10.5281/zenodo.21816594).

## Acknowledgments

This study was supported by funds from the 10.13039/501100001809National Natural Science Foundation of China (82070337, 82270395, and 82302458); the 10.13039/501100003399Science and Technology Commission of Shanghai Municipality (21ZR1440900); the 1 3·5 Project for Disciplines of Excellence–Clinical Research Incubation Project, West China Hospital, 10.13039/501100004912Sichuan University (no. 2019HXFH029); and the Science and Technology Project of Sichuan Province Health Commission, China (no. 21PJ035).

## Author contributions

J.W., Y.W., and W.L. analyzed data and wrote the paper. Y.Z. and Y.Q. designed the research. Zhengjie Wang, J.L., Zikun Wang, L.S., Y.P., Y.S., and L.Z. contributed to data collection and correction. Y.Z. and Y.Q. are responsible for the overall content as guarantors. All authors contributed to, processed, and edited the manuscript. All authors provided critical revisions of the manuscript and approved the final manuscript.

## Patient consent statement

The authors confirm that patient consent forms have been obtained for this article.

## Declaration of interests

The authors declare no competing interests.

## Declaration of generative AI and AI-assisted technologies in the writing process

AI-assisted tools were used only for language editing and improving the clarity of the manuscript. All scientific content, interpretations, and conclusions were developed by the authors and carefully reviewed and verified.

## STAR★Methods

### Key resources table

**Table:** 

REAGENT or RESOURCE	SOURCE	IDENTIFIER
**Antibodies**		

ITGA8 antibody	R&D Systems	Cat#MAB6194; RRID: AB_10551972
Vimentin antibody	Abcam	Cat#ab8978; RRID: AB_306907
CD31 antibody	Abcam	Cat#ab182981; RRID: AB_2920881
CD11b antibody	Abcam	Cat#ab8878; RRID: AB_306831
CD206 antibody	Abcam	Cat#ab64693; RRID: AB_1523910
α-SMA antibody	Proteintech	Cat#14395-1-AP; RRID: AB_2223009
cTnT antibody	ABclonal	Cat#A25223
DAPI	Sigma	Cat#D9542

**Bacterial and virus strains**		

AAV9 vector	Hanbio Biotechnology	pHBAAV-col1a2-ZsGreen

**Biological samples**		

Human left atrial appendage tissues from VAF patients and sinus rhythm donors	West China Hospital	This paper

**Chemicals, peptides, and recombinant proteins**		

DPBS	Thermo Fisher	Cat#14190144
DMEM	Corning	Cat#10-013-CV
Fetal bovine serum	Gemini	Cat#900-108
Penicillin-Streptomycin	Sigma	Cat#P4333
TRIzol	Invitrogen	Cat#15596018CN
Polybrene	Sigma	Cat#TR-1003
Puromycin	Beyotime	Cat#ST551-50 mg
4% Formalin	Servicebio	Cat#G1101

**Critical commercial assays**		

SHBIO Nuclei Isolation Kit	SHBIO	Cat#52009-10
Chromium Single Cell 3′ Library and Gel Bead Kit v3	10*x* Genomics	Cat#CG000204
Lentivirus Concentration Kit	Genomeditech	Cat#GM-040801

**Deposited data**		

Single-nucleus RNA sequencing dataset	NODE	OEP00006765; https://www.biosino.org/node/project/detail/OEP00006765
Analysis scripts	GitHub	https://github.com/YuliangWang316/AF_SinglecellRNAseq

**Experimental models: Cell lines**		

Human cardiac fibroblast cells (HCFCs)	BLUEFBIO	Cat#YS-01X7593
HEK293T cells	American Type Culture Collection	Cat#CRL-1573

**Experimental models: Organisms/strains**		

Rat: Sprague-Dawley rats	Scientific Research Platform of Shanghai Chest Hospital	Cat#R-SD-01

**Oligonucleotides**		

ITGA8 shRNA target sequence	This paper	5′-CCAGATAAGCAGGAGATAATT-3′

**Recombinant DNA**		

AAV9-Col1a2-ITGA8 shRNA	Hanbio Biotechnology	Cat#111012910
AAV9-Col1a2-NC control vector	Hanbio Biotechnology	Cat#111012909
pCMV.DR8 packaging plasmid	Addgene	Cat#8455
pMD2.G packaging plasmid	Addgene	Cat#12259

**Software and algorithms**		

Cell Ranger v3.0.1	10*x* Genomics	https://www.10xgenomics.com/support/software/cell-ranger
Seurat v3.0.2	Satija et al.	https://satijalab.org/seurat/
Harmony	Korsunsky et al.	https://github.com/immunogenomics/harmony
STAR aligner	Dobin et al.	https://github.com/alexdobin/STAR
Monocle v2.20.0	Trapnell laboratory	http://cole-trapnell-lab.github.io/monocle-release/
CellChat v1.6.1	Jin et al.	https://github.com/sqjin/CellChat
clusterProfiler	Yu et al.	https://bioconductor.org/packages/clusterProfiler
Msigdbr	MSigDB/R package	https://igordot.github.io/msigdbr/
DESeq2	Bioconductor	https://bioconductor.org/packages/DESeq2
Analysis scripts	Zenodo	doi: https://doi.org/10.5281/zenodo.21816594

**Other**		

Wheat germ agglutinin (WGA)	Invitrogen	Cat#W11261
Masson staining kit	Yeasen Biotechnology	Cat#60532ES74
Chromium Single Cell Processor	10*x* Genomics	N/A
NovaSeq 6000 sequencing system	Illumina	N/A
Mindray Z60 echocardiography system	Mindray	Cat#211397
BL-420N biological signal acquisition system	Chengdu Taimeng Technology	Cat#BL-420N
Transesophageal pacing electrode	N/A	4.5 Fr
Boyden chamber	Falcon	Cat#353097
QuantStudio 6 Flex Real-Time PCR System	Applied Biosystems	Cat#4485694
PowerUp SYBR Green Master Mix	Applied Biosystems	Cat#A25741
SuperScript First Strand Synthesis System	Vazyme	Cat#R211

### Experimental model and subject participant details

#### Human specimens

Left atrial appendage tissues were collected from three patients with persistent rheumatic valvular atrial fibrillation undergoing mitral valve replacement, maze ablation and left atrial appendage resection. For comparison, LAAs from 3 sinus rhythm (SR) subjects from the same region as well as 3 VAF patients were harvested from healthy donors matched by age and gender. The healthy specimens, which were obtained from the homograft bank, had been rejected for clinic use and therefore allocated for research. The clinical criteria for the healthy donors rejected for transplantation included the failure in pairing or the recipient had been already evaluated not suitable for receiving transplantation. The study was approved by the Ethics Committee Board of West China Hospital, Sichuan University (approval number 2018301).

#### Animal model

Male Sprague-Dawley rats (250–300 g) were used. Animals were randomly assigned into control, AF, AF + AAV9-Col1a2-NC, and AF + AAV9-Col1a2-ITGA8 shRNA groups. Animal experiments were approved by the Animal Ethics Committee of Shanghai Jiao Tong University (KS26044).

### Method details

#### Sample preparation and single-nucleus RNA sequencing

Tissues were washed with DPBS, minced on ice, and nuclei were isolated using SHBIO Nuclei Isolation Kit. Libraries were generated using Chromium Single Cell 3′ Library and Gel Bead Kit v3 (10*x* Genomics), processed using Chromium Single Cell Processor, and sequenced using Illumina NovaSeq 6000.

#### Read alignment and transcriptomic processing

FASTQ files were processed using Cell Ranger v3.0.1. Reads were aligned to the human GRCh38 genome using STAR. Gene-barcode matrices were analyzed using Seurat v3.0.2 with Harmony integration.

#### Cell clustering and annotation

Dimensional reduction was performed using PCA and UMAP. Cell clusters were annotated using established marker genes. Differential expression analysis was performed using Seurat, DESeq2 and pseudo-bulk approaches.

#### Functional enrichment analysis

Pathway enrichment analyses were performed using clusterProfiler and msigdbr based on MSigDB gene sets.

#### Trajectory analysis

Trajectory inference was performed using Monocle v2.20.0 with DDRTree dimensionality reduction.

#### Cell-cell communication analysis

Cell-cell communication analysis was performed using CellChat v1.6.1.

#### Histology and immunostaining

Human and rat cardiac tissues were processed for H&E staining, Masson’s trichrome staining, WGA staining, and immunostaining using antibodies against ITGA8, Vimentin, CD31, CD11b, CD206, α-SMA and cTnT.

#### Cell culture and migration assay

Human cardiac fibroblast cells were cultured in DMEM containing fetal bovine serum and penicillin-streptomycin. Migration was assessed using Boyden chamber assays.

#### Lentiviral gene manipulation

Lentiviral vectors were generated in HEK293T cells using pCMV.DR8 and pMD2.G packaging plasmids. Cells were infected in the presence of polybrene and selected using puromycin.

#### AAV-mediated ITGA8 knockdown

AAV9 vectors carrying Col1a2 promoter-driven ITGA8 shRNA or control sequence were obtained from Hanbio Biotechnology and administered by tail-vein injection.

#### Echocardiography and electrophysiology

Cardiac function was evaluated using Mindray Z60 echocardiography. AF inducibility was assessed using transesophageal pacing and BL-420N biological signal acquisition system.

#### RT-qPCR

RNA was extracted using TRIzol. cDNA synthesis and qPCR were performed using SuperScript First Strand Synthesis System, PowerUp SYBR Green Master Mix, and QuantStudio 6 Flex Real-Time PCR System.

### Quantification and statistical analysis

Data are presented as mean ± SEM. Statistical tests and biological replicate numbers are indicated in figure legends. Two-group comparisons used unpaired two-tailed Student’s *t* test. Multiple-group comparisons used one-way ANOVA with Tukey correction. AF inducibility was analyzed using chi-square or Fisher’s exact test. Statistical significance was defined as ∗*p* < 0.05; ∗∗*p* < 0.01; ∗∗∗*p* < 0.001; ∗∗∗∗*p* < 0.0001, and ns, not significant.

### Additional resources

This study was registered at the Chinese Clinical Trial Registry under registration number ChiCTR1800019870.
