# Supplementary material for: Single nucleus transcriptomics reveals ITGA8-driven fibroblast remodeling in rheumatic valvular atrial fibrillation
Source: iScience. 2026 Sep 1;29(9):117310. doi: 10.1016/j.isci.2026.117310 (PMC13571147; doi:10.1016/j.isci.2026.117310)
Supplement: Supplementary file 1 [file mmc1.pdf]

iScience, Volume *29*

Supplemental information

Single nucleus transcriptomics reveals ITGA8-driven fibroblast remodeling in rheumatic valvular atrial fibrillation

Jinjin Wu, Yuliang Wang, Weizhuo Liu, Zhengjie Wang, Jingjing Liu, Zikun Wang, Lei Yang, Lin Sun, Yilin Pan, Yangyang Sun, Li Zhang, Yongjun Qian, and Yangyang Zhang

Supplementary Figure 1

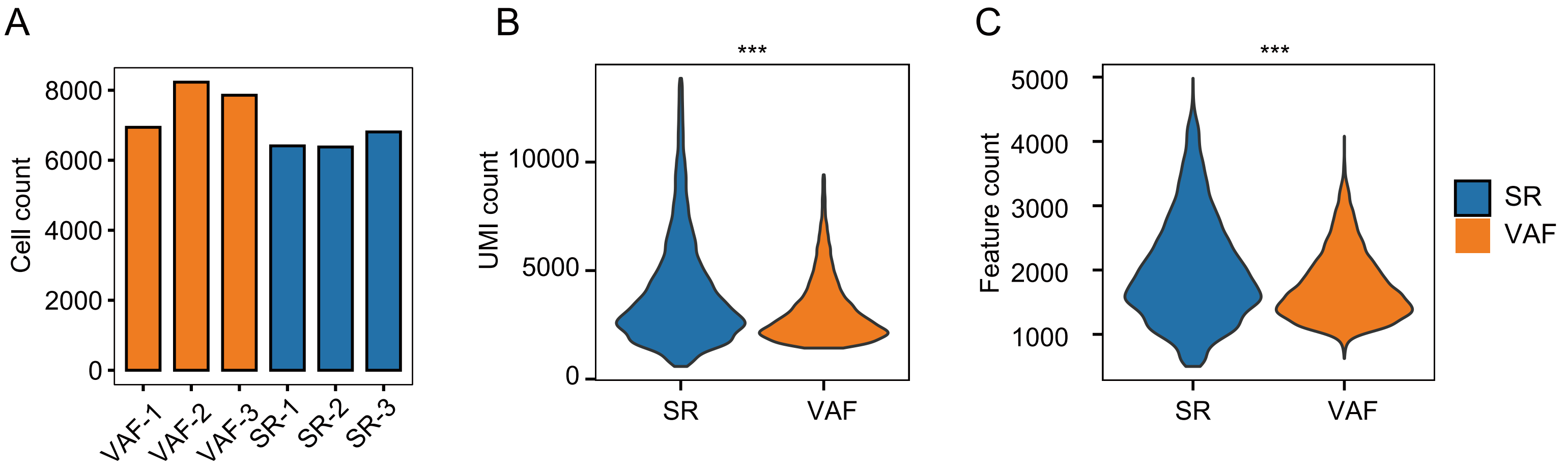

Supplementary Figure 1. Quality measurements of different samples

Quality measurements of different data modalities and samples. Included are the number of nucleuses per sample (A), number of unique molecular identifiers (UMIs)(B), and number of features(C). Different group are represented in different colors, VAF (orange) to SR (blue). Our study integrated data from both healthy and VAF patients. The t-test was used for comparisons between two groups, $P < 0.05$ was considered as statistical significance in this study. *$P < 0.05$; **$P < 0.01$, ***$P < 0.001$.

Supplementary Figure 2

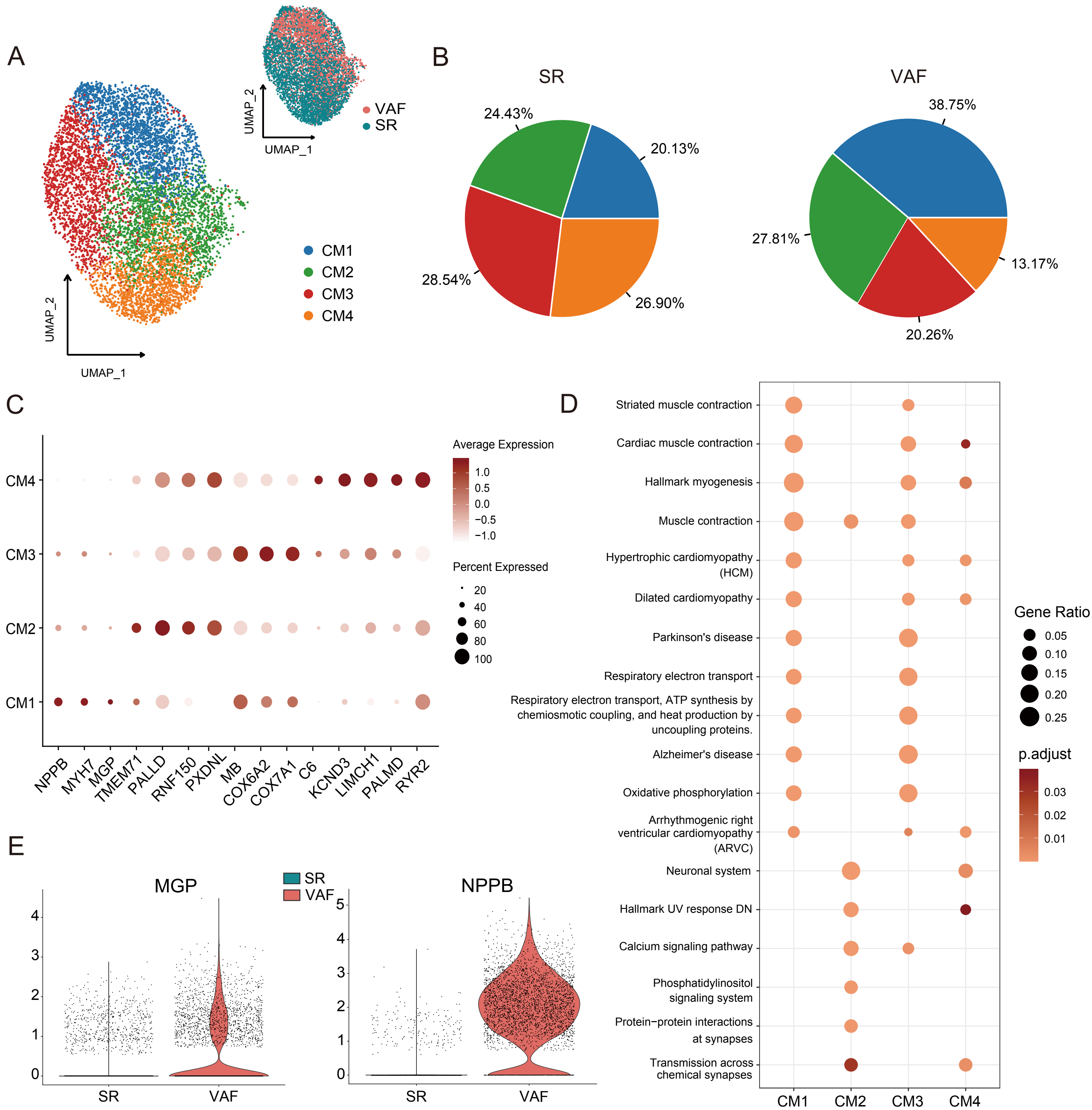

Supplementary Figure 2. Exploration of cardiomyocyte subsets in SR and VAF

A. the four cardiomyocytes (CM1-CM4) subsets identified by the subclustering of cardiomyocytes from SR and VAF hearts. The inset (right-top) showed the cardiomyocytes corresponding to different disease states, SR (green) and VAF (red). **B**. the proportions of CM1-CM4 relative to all cardiomyocytes within SR (left) and VAF (right). **C**. the gene signatures that distinguish cardiomyocyte states (genes selected by Seurat differential expression analysis). As for cardiomyocytes sub-clustering, we first subset cardiomyocytes cluster, then we re-normalized, re-scaled, re-PCA and re-clustering. Then, we used NPPB, MYH7, MGP, TMEM71, PALLD, RNF150, PXDNL, MB, COX6A2, COX7A1, C6, KCND3, LIMCH1, PALMD and RYR2 as marker genes for sub-clustering. **D**. pathways enriched by the differentially expressed genes in CM1-CM4. GeneRatio indicates the number of genes mapped to each pathway and color indicates the adjusted *P* value from the pathway enrichment analysis. **E**. the violin plots depict the RNA expression of MGP and NPPB in SR (blue) and VAF (red) cardiomyocytes. Our study integrated data from both healthy and VAF patients.

Supplementary Figure 3

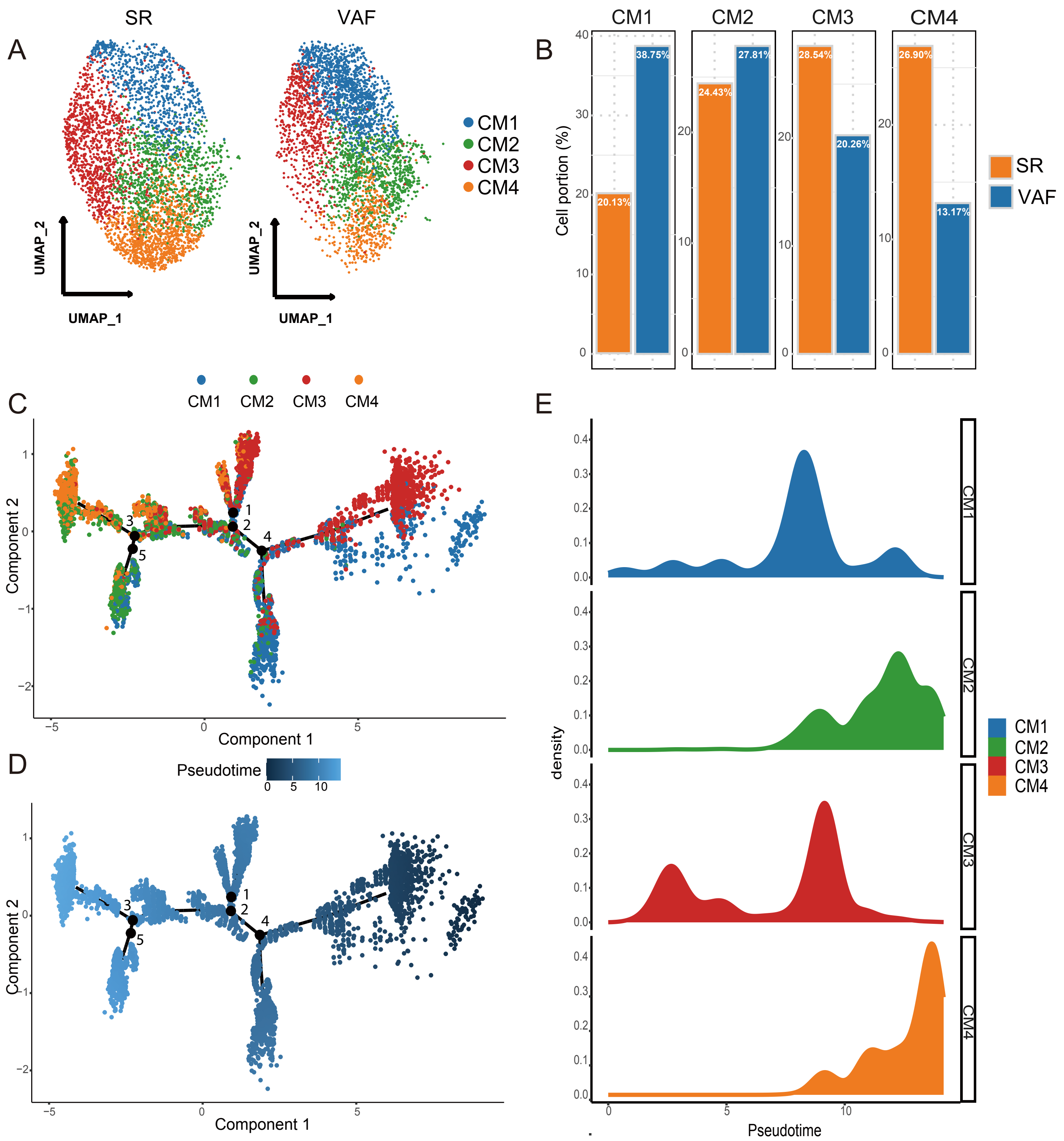

Supplementary Figure 3. Cell distribution and pseudotime of cardiomyocyte subsets in SR and VAF

A. Distribution of the four cardiomyocytes subsets within VAF (left) and SR (right). **B**. Bar plots showing the proportion of four cardiomyocytes populations in VAF (blue) and SR (orange). Monocle2 trajectory analysis of fibroblasts colored by fibroblast subsets (**C**) and pseudotime (**D**). **E**. the density curves of different subsets of cardiomyocytes subsets over the pseudotime. Our study integrated data from both healthy and VAF patients.

Supplementary Figure 4

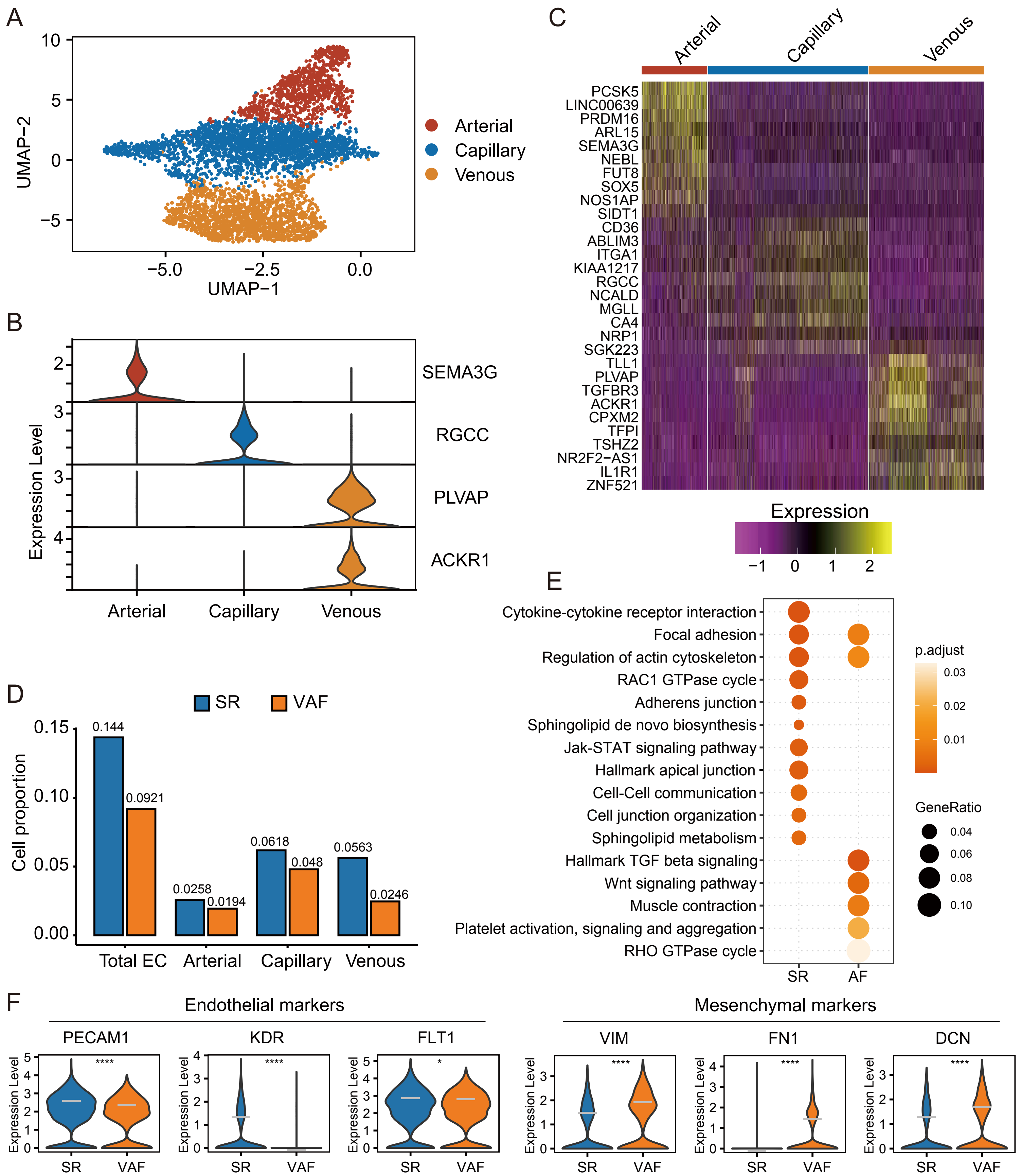

Supplementary Figure 4. Subclustering of vascular EC and the functional changes of vascular EC subsets in the development of VAF pathology

A. the three vascular endothelial cell subsets identified by subclustering of vascular ECs from SR and VAF hearts. **B**. the violin plots depict the marker genes of the three vascular endothelial cell subsets. As for endothelial cells sub-clustering, we first subset endothelial cells clustering, then we re-normalized, re-scaled, re-PCA and re-clustering. Then, we used SEMA3G, RGCC, PLVAP and ACKR1 as marker genes for sub-reclustering. **C**. the expression patterns of key genes within distinct subsets of vascular endothelial cells. **D**. the relative proportions of arterial, capillary, venous, and total endothelial cells in comparison to the total cell counts identified by snRNA-seq for the comparison of individuals with VAF (orange) to SR (blue). **E**. Pathways enriched by DEGs within SR and VAF samples. GeneRatio represents the ratio of DEGs in a specific pathway to the total number of genes in that pathway. The color indicates the adjusted *P* value by the pathway enrichment analysis. **F**. Violin plots depict the expression of endothelial markers (PECAM, KDR, and FLT1) and mesenchymal markers (VIM, FN1, and DCN) in endothelial cells from SR (shown in blue) and individuals with VAF (shown in orange). The t-test was used for comparisons between two groups, $P < 0.05$ was considered as statistical significance in this study. *$P < 0.05$; **$P < 0.01$, ***$P < 0.001$. Our study integrated data from both healthy and VAF patient.

Supplementary Table 1

Baseline characteristics of three VAF patients.

	1	2	3
**Sex**	Male	Male	Male
**Age (years)**	54	51	53
**Weight (kg)**	52	63	64
**Height (cm)**	160	164	172
**Operation**	MVR+TVP+MAZE+LAA Resection	MVR+TVP+MAZE+LAA Resection	MVR+MAZE+LAA Resection
**AF-type**	Persistent	Persistent	Persistent
**AF duration (year)**	4	3	4
**NYHA classification**	Class II	Class III	Class II
**Diabetes**	No	No	No
**Hypertension**	No	No	No
**Hyperlipidemia**	No	No	No
**Myocardial infarction**	No	No	No
**Congenital heart disease**	No	No	No
**Cardiomyopathy**	No	No	No
**LVEF (%)**	65	55	57
**LAD (mm)**	50	52	52
**LAD/BSA (mm/m^2)**	33.6	31.4	30.3

**MV disease**	Severe MS, rheumatic	Severe MS, rheumatic	Severe MS, rheumatic
**CHA_2DS_2-VASc Scores**	0	0	0

VAF = rheumatic valvular AF-associated remodeling (VAF); MVR = Mitral valve replacement; TVP = Tricuspid valvuloplasty; LAA = Left atrial appendage; NYHA = New York Heart Association; LVEF = Left ventricular ejection fraction; LAD = Left atrial diameter; BSA = Body surface area; MV = Mitral valve; MS = Mitral stenosis
